# Giant Magnetoresistance in a Chemical Vapor Deposition Graphene Constriction

**DOI:** 10.1021/acsnano.1c09815

**Published:** 2022-02-03

**Authors:** Luke W. Smith, Jack O. Batey, Jack A. Alexander-Webber, Yu-Chiang Hsieh, Shin-Jr Fung, Tom Albrow-Owen, Harvey E. Beere, Oliver J. Burton, Stephan Hofmann, David A. Ritchie, Michael Kelly, Tse-Ming Chen, Hannah J. Joyce, Charles G. Smith

**Affiliations:** † Department of Physics, Cavendish Laboratory, University of Cambridge, Cambridge CB3 0HE, United Kingdom; ‡ Department of Physics, National Cheng Kung University, Tainan 701, Taiwan; ¶ Electrical Engineering Division, Department of Engineering, University of Cambridge, Cambridge CB3 0FA, United Kingdom; § Center for Quantum Frontiers of Research & Technology (QFort), National Cheng Kung University, Tainan 701, Taiwan

**Keywords:** multiplexed device arrays, graphene, CVD, giant magnetoresistance, magnetotransport, Fowler-Nordheim tunneling, direct tunneling

## Abstract

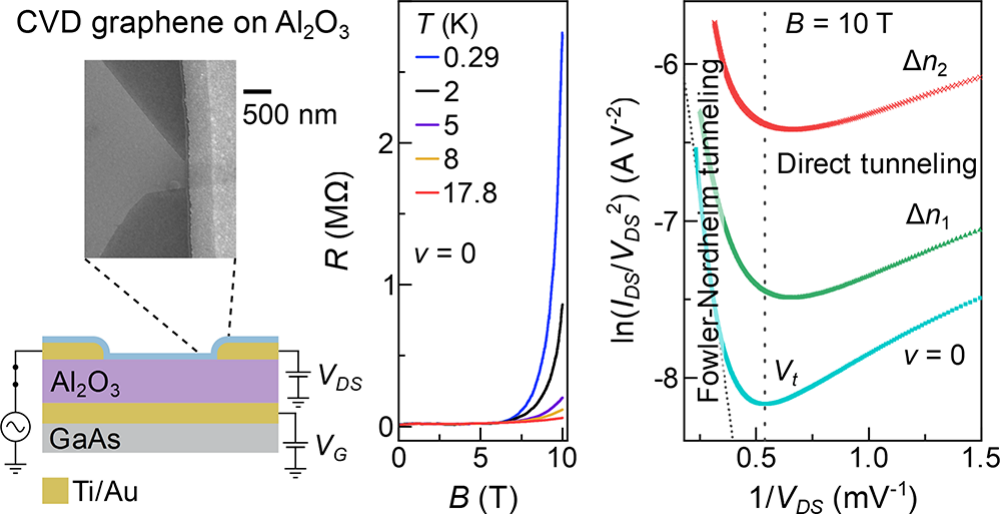

Magnetic field-driven insulating states in graphene are associated with samples of very high quality. Here, this state is shown to exist in monolayer graphene grown by chemical vapor deposition (CVD) and wet transferred on Al_2_O_3_ without encapsulation with hexagonal boron nitride (*h*-BN) or other specialized fabrication techniques associated with superior devices. Two-terminal measurements are performed at low temperature using a GaAs-based multiplexer. During high-throughput testing, insulating properties are found in a 10 μm long graphene device which is 10 μm wide at one contact with an ≈440 nm wide constriction at the other. The low magnetic field mobility is ≈6000 cm^2^ V^–1^ s^–1^. An energy gap induced by the magnetic field opens at charge neutrality, leading to diverging resistance and current switching on the order of 10^4^ with DC bias voltage at an approximate electric field strength of ≈0.04 V μm^–1^ at high magnetic field. DC source–drain bias measurements show behavior associated with tunneling through a potential barrier and a transition between direct tunneling at low bias to Fowler-Nordheim tunneling at high bias from which the tunneling region is estimated to be on the order of ≈100 nm. Transport becomes activated with temperature from which the gap size is estimated to be 2.4 to 2.8 meV at *B* = 10 T. Results suggest that a local electronically high quality region exists within the constriction, which dominates transport at high *B*, causing the device to become insulating and act as a tunnel junction. The use of wet transfer fabrication techniques of CVD material without encapsulation with *h*-BN and the combination with multiplexing illustrates the convenience of these scalable and reasonably simple methods to find high quality devices for fundamental physics research and with functional properties.

Insulating phases in graphene at high magnetic fields are typically observed only in exceptionally high quality devices, characterized by high carrier mobility and low residual carrier density. Demonstrations have been almost exclusively limited to exfoliated graphene flakes^1−6^ and, recently, ultrahigh mobility graphene^7,8^ grown by chemical vapor deposition (CVD) and encapsulated with hexagonal boron nitride (*h*-BN).^9^ These devices are fabricated from monolayer material using sophisticated methods that limit scalability such as the use of exfoliated material, suspension of the graphene,^10,11^ fabrication on *h*-BN substrates,^12,13^ or encapsulation with *h*-BN.^9,14,15^ At a high perpendicular magnetic field, the 2D density of state becomes quantized in Landau levels with energy , where *N* is the Landau level index, *v*_F_ is the Fermi velocity, *ℏ* is the reduced Planck constant, and *e* is the electron charge, and an insulating phase can exist at charge neutrality^3,14^ characterized by a resistance that diverges with *B* as a magnetic field-dependent energy gap opens in the *N* = 0 Landau level. Here, we find such an insulating state can exist in conventionally wet transferred monolayer CVD graphene devices fabricated on amorphous oxides.

The device is contained within an array of CVD graphene devices nominally patterned as 10 μm-wide squares measured using a multiplexer^16^ at cryogenic temperatures. Multiplexing is a means for high-throughput testing and characterization of many devices,^17−19^ and the large number tested can lead to the discovery of individual devices with exceptional qualities that can be opportunistically studied. Here, a narrow constriction of ≈440 nm is created at one end of the graphene square, apparently by graphene tearing during fabrication. The resistance at charge neutrality diverges with *B* showing insulating behavior and an energy gap that increases with *B*. The conductivity appears to be reasonably temperature-independent below ≈2 K, and an increase in *T* shows activated behavior from which the energy gap is estimated to be ∼2.4–2.8 meV at *B* = 10 T. This behavior suggests the existence of a region of high electronic quality within the narrow channel, which dominates transport at high *B*. The analysis of charge transport mechanisms using DC source–drain bias measurements and quantum tunneling models^20−24^ at low *T* shows a transition from direct tunneling to Fowler-Nordheim tunneling with increasing DC bias from which the length of the tunneling region is estimated to be on the order of ≈100 nm. The device shows the current switching of order ∼10^4^ with DC source–drain bias for an approximate electric field strength of ∼0.04 V μm^–1^.

Here, although the narrow constriction is unintentionally created, it is straightforward to create the same geometry deliberately. A second device is fabricated with a similar contact configuration and a 600 nm-wide constriction defined next to one contact by electron–beam lithography. Similar behavior is observed in terms of an insulating state although being impacted by lithographic patterning and doping, potentially indicating the quality of the constriction edge as an important consideration. We speculate that less disorder is present in the unintentional constriction due to its formation as a result of tearing, where edges are never exposed to plasma etching, which can cause greater edge disorder and contamination compared to cleaner alternatives^25,26^ thus motivating further studies toward developing clean processes for constriction edges such as *h*-BN etch masks.^27,28^ The observation of the insulating phase at charge neutrality in wet transferred CVD graphene on amorphous oxide suggests the intriguing potential of developing systems to investigate exotic quantum phenomena using conventional and scalable fabrication techniques. A primary application of multiplexing is the statistical analysis of large data sets and identifying trends in data ensembles.^16,29,30^ Statistical analysis identifies general behavior, allowing outliers to be selected and studied in detail. Our present work highlights the capability of our multiplexing system for an in depth study of the physics of such channels using numerous techniques including magnetic field, source–drain bias, and temperature from which one can obtain a detailed understanding of the fundamental phenomena. The identification of optimal devices has been a bottleneck in studies of fundamental physics, since multiple devices usually must be measured before a suitable one is found. The multiplexer expedites this process in a much more efficient manner. Our study also highlights the ability of the multiplexer to drive device development, since the inherent inhomogeneity in nanostructure devices leads to these outliers that can have desirable properties. The study of the outliers reveals a particular set of device conditions creating the observed behavior, and the identification of differences from other devices in the array provides information needed to reproduce the behavior deterministically.

## Results and Discussion

The multiplexer is fabricated on a GaAs/AlGaAs heterostructure containing a two-dimensional electron gas (2DEG) with a conducting path from one input to 16 outputs. Individual graphene devices are connected to each output and selected using addressing gates. Multiplexer operation, fabrication, and layout are described in ref (16). The device measured here is different from that in ref (16) where the emphasis was on the versatility of the multiplexer for high-throughput testing and compatibility with diverse nanoelectronic devices *via* integration of arbitrary nanomaterials including 2D materials (both CVD-grown films and exfoliated flakes) and semiconductor nanowires.^16^ The multiplexing platform can also be adapted for devices fabricated within a 2DEG and controlled by surface gates.^19,29−33^

**Figure 1:**
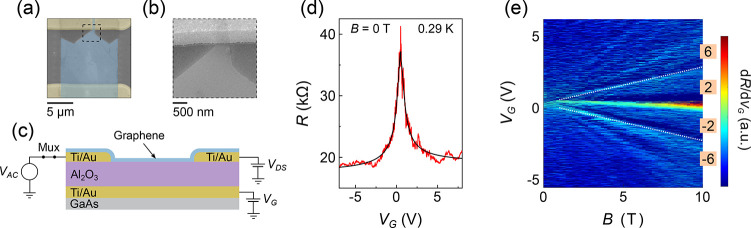
(a) False color scanning electron microscopy image of the graphene device. Graphene and source–drain contacts are shown in blue and yellow, respectively. (b) Zoomed image of the dashed rectangular area in (a). (c) Schematic cross section from source to drain. The switch indicates multiplexer control circuitry.^16^ (d) The red curve shows transfer characteristics as a function of back gate voltage at *B* = 0 T and *T* = 0.29 K. The black curve shows a fit using eq 1. (e) Magnetic field dependence of d*R*/d*V*_G_ as a function of *V*_G_. Filling factors ν = ±2 and ±6 are labeled, and white dotted lines highlight local resistivity maxima corresponding to half filled ±1 Landau levels.

Here, the array of graphene devices is patterned in a monolayer CVD graphene film after wet transfer^34^ to the multiplexer host chip, as described in the Methods. Scanning electron microscopy images of the measured device are shown in Figure 1a,b. The image shows a narrow constriction next to a contact, and the region around the constriction appears to have reasonably well-defined edges and angles, suggesting the graphene constriction was formed by tearing along clear crystallographic directions. Tearing has been observed to give armchair or zigzag termination,^25^ which could indicate the constriction edges are atomically clean and feature minimal dangling bonds and scattering sites.^26^ The constriction is ∼440 nm at the narrowest point. Figure 1c shows a schematic cross section through the device (side profile). Source–drain contacts are created prior to graphene transfer, and 95 nm thick Al_2_O_3_ is deposited by atomic layer deposition above the back gate. The two-terminal differential conductance is measured in a ^3^He cryostat.

### Transport Characterization

Figure 1d shows resistance (*R*) as a function of back gate voltage (*V*_G_) at *B* = 0 T and *T* = 0.29 K. The black curve is a fit to the data using^35,36^
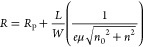
1where *R*_P_ is the parasitic resistance, *L*/*W* is the length to width ratio, μ is the device mobility, *n*_0_ is the residual carrier density, and *n* is the back gate-dependent carrier density. The density is given by *n* = *C*_G_(*V*_G_ – *V*_0_)/*e*, where *C*_G_ is the gate capacitance per unit area *C*_G_ = ϵ_0_ϵ/*t*_ox_, *V*_0_ is the charge neutrality-point voltage, ϵ_0_ is the free space permittivity, ϵ is the oxide permittivity, and *t*_ox_ is the oxide thickness.

The mobility and parasitic resistance are estimated separately for electrons and holes, *i.e.*, for *V* < *V*_0_ (*V* > *V*_0_), μ = μ_hole_, and *R*_P_ = *R*_P,hole_ (μ = μ_electron_ and *R*_P_ = *R*_P,electron_). For this equation, *V*_0_ is obtained by initially fitting data with density-independent *R*_P_ and μ, giving 0.52 V. Fitted parameters are *R*_P,hole_ = 17.4 kΩ, *R*_P,electron_ = 19.0 kΩ, *n*_0_ = 1.3 × 10^11^ cm^–2^, μ_hole_ = 5350, and μ_electron_ = 6360 cm^2^ V^–1^ s^–1^ for *L*/*W* ≈ 2.3. *L*/*W* is estimated by dividing the conducting area into squares and analyzing the resulting resistor network to give an equivalent length-to-width ratio.

Figure 1e shows the Landau level splitting with the magnetic field^37−39^ with d*R*/d*V*_G_ plotted as a function of *B* and *V*_G_. Local resistivity maxima corresponding to where ±1 Landau levels are half filled are indicated by dotted white lines, and regions between correspond to quantum Hall plateaus. The gate capacitance is estimated from the gate dependence of quantum Hall states ν = ±2, where ν is the filling factor, using d*n*/d*B* = *νe*/*h* and d*n*/d*V*_G_ = *C*_G_/*e*, giving *C*_G_ ≈ 60 nF cm^–2^ and ϵ = 6.4. Landau level splitting also provides an alternative estimate of μ using the approximation of μ ≈ 1/*B* at *B* where Landau levels can be resolved.^40^ This gives a possible mobility up to μ ≈ 8000 cm^2^ V^–1^s^–1^, as discussed in Figure S3a, and mean free path *l* = *v*_F_τ ≈ 17 nm using scattering time τ ≈ 1/ω_c_, cyclotron frequency , and *v*_F_ = 1 × 10^6^ m s^–1^.

### Magnetoresistance Measurements

**Figure 2:**
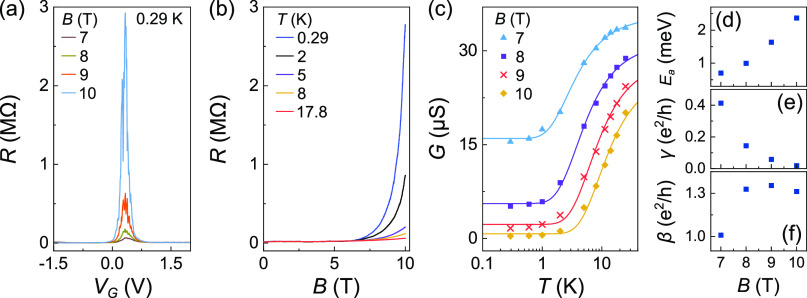
(a) Resistance as a function of back gate voltage (*V*_G_) for *B* = 7, 8, 9, and 10 T at *T* = 0.29 K. (b) Resistance as a function of magnetic field for different temperatures at the ν = 0 insulating state, measured at *V*_G_ = 0.31 V. (c) Conductance *G* at ν = 0 as a function of log_10_*T* for *B* = 7, 8, and 9, and 10 T. Solid lines show fits to the data using the Fermi–Dirac function. The fitting parameters using eq 2, *i.e.*, energy gap *E*_a_, fitted conductance at zero temperature γ, and pre-exponential factor β, are shown in panels (d), (e), and (f), respectively.

**Figure 3:**
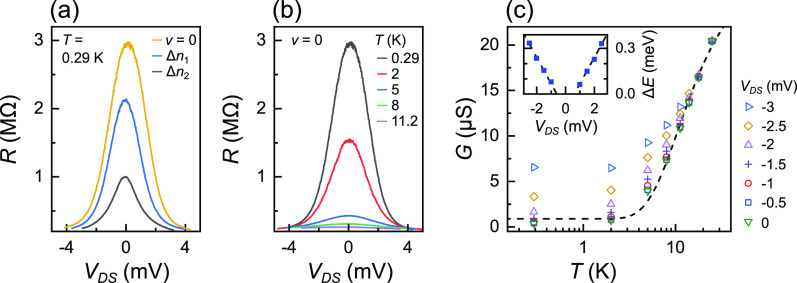
DC source–drain bias measurements in the ν = 0 insulating state. Magnetic field (*B*) = 10 T for all data in (a)–(c). (a) Resistance as a function of DC source–drain bias at ν = 0, Δ*n*_1_ = 2.2 × 10^10^ cm^–2^, and Δ*n*_2_ = 4.5 × 10^10^ cm^–2^, where Δ*n* represents the change in density from ν = 0. Temperature *T* = 0.29 K. (b) Temperature dependence at ν = 0. (c) Conductance *G* at ν = 0 for select *V*_DS_ as a function of *T* after subtracting series resistance. The dashed line is a fit at *V*_DS_ = 0 mV using eq 2. The inset shows Δ*E* = *k*_B_Δ*T* where Δ*T* is the temperature difference between data points at *T* = 2 K and the same conductance on the *V*_DS_ = 0 mV fit line. Dashed lines show linear fits with the same absolute value of gradient for positive and negative *V*_DS_.

Figure 2a shows *R* as a function of *V*_G_ at *B* = 7, 8, 9, and 10 T and *T* = 0.29 K. The resistance at charge neutrality increases by almost 3 MΩ at *B* = 10 T. Figure 2b shows *R* at *V*_G_ = 0.31 V as a function of *T*, which diverges with *B* for the lowest *T*. The estimated *B*-dependent resistance of the GaAs multiplexer is subtracted from the data in Figure 2 as described in Figure S1. Data in Figure 2b,c are measured at *V*_G_ = 0.31 V. Data for Figure 3 are obtained at *V*_G_ = 0.32 V after reoptimization. Both are taken to represent the high *B* insulating state at ν = 0 when discussing each figure. Fitting transfer characteristics at *B* = 0 T using eq 1 estimates a charge neutrality point voltage of 0.52 V. Differences may arise since the low *B* value is given by fitting methods over a broad range of *V*_G_, whereas values at *B* = 10 T are experimentally determined to be as close as possible to the maximum *R*. Additionally, differences may conceivably arise since the low *B* characteristics likely represent an average of the entire graphene area, whereas at high *B* a small region within the constriction appears to dominate transport characteristics.

The conductance *G* at *V*_G_ = 0.31 V is plotted as a function of *T* on a lin-log scale in Figure 2c for *B* = 7, 8, 9, and 10 T. This behavior becomes visible for *B* ∼ 7 T and above. Similar to that in ref (1), *G* appears to become relatively *T* independent at the lowest temperatures measured. Solid lines show fits to the data for activated transport using a Fermi–Dirac distribution^15,41^
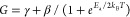
2where *E*_a_ = 0.7, 1.0, 1.6, and 2.4 meV is the magnetic field-dependent energy gap at *B* = 7, 8, 9, and 10 T, respectively, *k*_B_ is the Boltzmann constant, γ is the zero *T* conductance, and β is a pre-exponential factor. Fits at each *B* are performed independently with *E*_a_, γ, and β as fitting parameters. Data are first fit using the Arrhenius equation, which gives energy gaps comparable to the thermal energy *k*_B_*T* at higher *T* values for which the measurement is performed (up to *T* = 25 K); therefore, the Fermi–Dirac function is used instead. Fitting using the Arrhenius equation is shown in Figure S2b.

The energy gap is shown as a function of *B* in Figure 2d. If a linear dependence *E*_a_ = Δ*E*_Z_ – Γ is assumed,^5,15^ where Δ*E*_Z_ is Zeeman energy *g*μ_B_*B*, μ_B_ is the Bohr magneton, and Γ is the disorder broadening of Landau levels, parameters Γ = 39.2 K and effective Landé *g*-factor *g* ≈ 10 are enhanced above the free-electron *g*-factor *g* = 2, as seen previously for exfoliated graphene,^2,13^ and attributed to exchange interactions.^15,42^ The quantum lifetime^5,43^ estimated from Landau level broadening  is reasonably comparable to scattering lifetimes estimated from mobility using the Drude formalism τ = (*h*μ/2*ev*_F_)(*n*/π)^1/2^ ≈ 105 fs at *n* = 2 × 10^12^ cm^–2^. However, the range of data in Figure 2d is too small to give a true indication of the relationship between *E*_a_ and *B*. An approximately linear relationship appears in some devices described in the literature^2,13^ but not necessarily in others.^10^ Reference (14) shows a linear relationship for a device engineered to minimize the Coulomb interaction and a square root dependence consistent with a Coulomb energy relationship in a separate device without this reduction. The estimated gap sizes here are much smaller than the Coulomb energy *E*_C_ = *e*^2^/4πϵ_0_ϵ*l*_B_ (*E*_C_ = 28 meV at *B* = 10 T), where *l*_B_ is the magnetic length . Previous studies show either *E*_a_ to be small^2^ compared to *E*_C_ or that they are similar values.^13^

Fitting parameters γ and β are shown in Figure 2e,f, respectively. The γ value describes *G* in the low *T* limit and nears zero as *B* increases. In a scenario of transport dominated by a region of gapped graphene at high *B*, this may represent hopping transport that diminishes as the energy gap *E*_a_ increases and the size of the wave function reduces with *B*, leading to less hopping between sites. The pre-exponential factor β is reasonably similar for *B* = 8, 9, and 10 T for fits performed independently at each field and is reduced at lower *B* as the gap becomes small. The β term may be related to the geometry of the gapped region, becoming reasonably constant above a crossover field where the gap opens. The insulating behavior does not occur in other devices in the multiplexed array that do not contain a narrow constriction. The previous observation of insulating states in graphene associated with high electronic quality devices supports a scenario where a high quality region exists within the constriction and dominates the transport characteristics at high *B*.

### DC Source–Drain Bias Measurements

The insulating state is further investigated by applying a DC source–drain bias across the graphene device (*V*_DS_). Figure 3a shows *R* as a function of *V*_DS_ at select *V*_G_ around charge neutrality for *T* = 0.29 K and *B* = 10 T. Data representing the high *B* insulating state at ν = 0 are obtained at *V*_G_ = 0.32 V; data at other *V*_G_ are labeled according to Δ*n* = *C*_G_(*V*_G_ – 0.32)/*e*. Supporting Information describes how *V*_DS_ across the graphene is extracted from the total source–drain bias applied to the circuit (*V*_B_), accounting for series resistance. The temperature dependence at ν = 0 is shown in Figure 3b. Energy scales associated with DC bias are reasonably similar to *E*_a_ from Fermi–Dirac fitting, *i.e.*, in Figure 3b, the full width at half-maximum (fwhm) of ≈3 mV at *T* = 0.29 and 2 K, accounting for a vertical offset from series resistance (*R*_S_) of ≈219 kΩ. Data in Figure 3a,b are plotted before subtracting *R*_S_. There is a small effective gating of the graphene at high *V*_DS_ due to the source–drain bias, discussed in Figure S4; however, the effect is negligible.

Figure 3c shows *G* as a function of *T* at select *V*_DS_ after subtracting *R*_S_. For clarity, data are shown for *V*_DS_ ≤ 0, and data for *V*_DS_ > 0 are similar. The dashed line shows a fit to *V*_DS_ = 0 mV data using the Fermi–Dirac distribution. Parameters are as follows: *E*_a_ = 2.8 meV, α = 0.02 *e*^2^/*h*, and β = 1.4 *e*^2^/*h*. Differences from the parameters estimated for *B* = 10 T data in Figure 2c may arise from reoptimizing *V*_G_ to find the maximum *R*. Data for finite *V*_DS_ appear to fall along the *V*_DS_ = 0 mV fit line at high *T* and diverge as *T* is reduced, becoming reasonably *T* independent for *T* < 2 K. The gap Δ*T* between *T* = 2 K data points for finite *V*_DS_ and the *V*_DS_ = 0 mV fit line is shown in the inset converted to energy Δ*E* = *k*_B_Δ*T*. Data at *T* = 2 K are chosen since *G* becomes reasonably *T* independent below this *T*. This may represent an equivalent thermal energy or an equivalent reduction in barrier height that would be required at *V*_DS_ = 0 mV to achieve the same effect. Dashed lines show linear fits with the same gradient (absolute value) for *V*_DS_ > 0 and *V*_DS_ < 0 mV, which cross the horizontal axis at |*V*_DS_|≈ 0.6 mV, similar to the AC excitation voltage *V*_AC_ = 400 μV (rms). The relationship appears linear up to bias voltages similar to the activation energy. We do not observe a power law relationship between Δ*T* and applied power at each *V*_DS_, which could be expected if Δ*T* represented a rise in electron temperature.^44^ This suggests the main impact of *V*_DS_ within this regime is to change the bias across the barrier. However, this is better investigated by analyzing the current–bias voltage characteristics.

### *I*–*V* Characteristics

**Figure 4:**
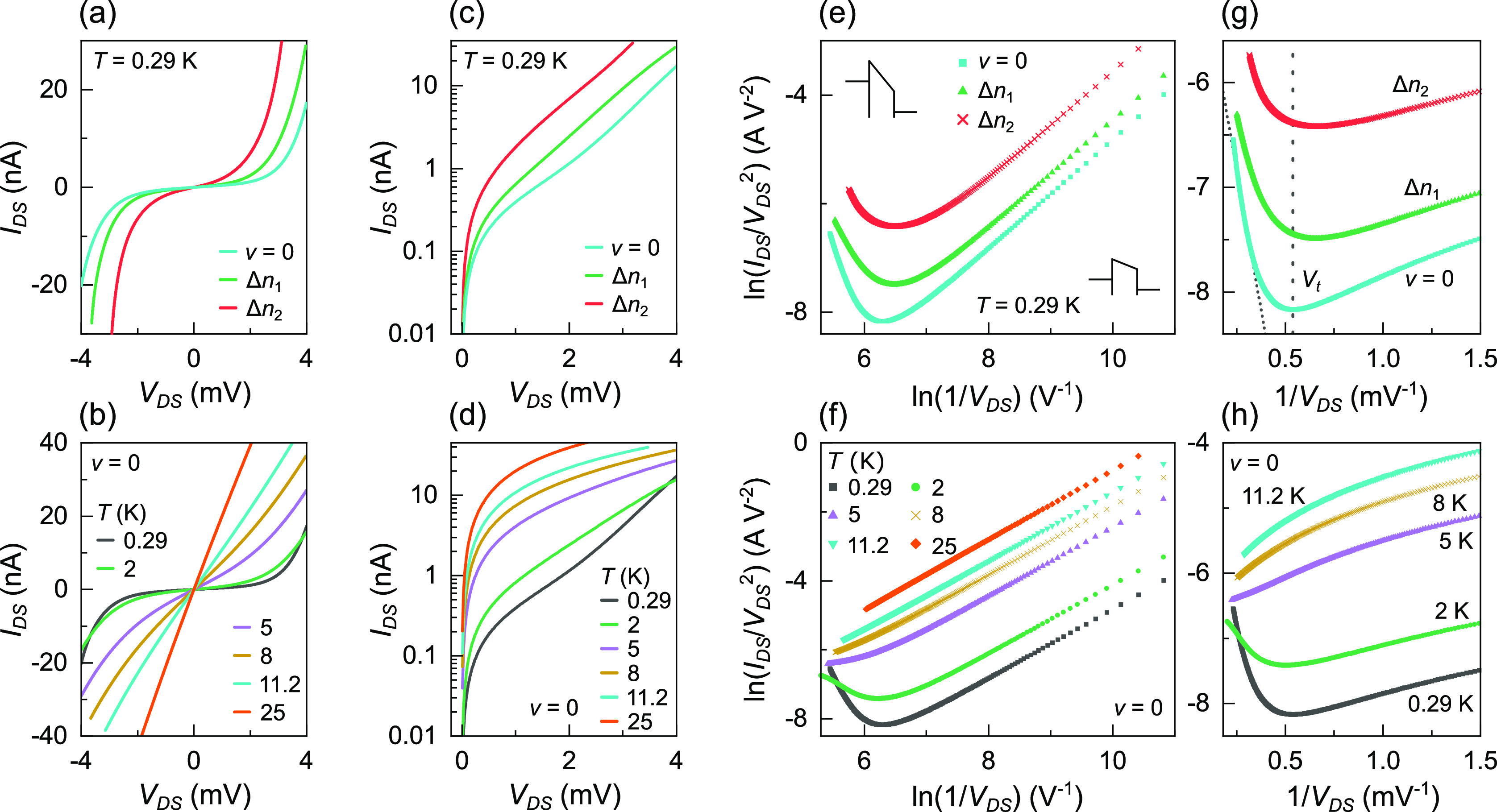
DC current–voltage characteristics. Magnetic field *B* = 10 T for all data in (a)–(h). The top row of (a), (c), (e), and (g) show data at ν = 0, Δ*n*_1_ = −2.2 × 10^10^ cm^–2^, and Δ*n*_2_ = −4.5 × 10^10^ cm^–2^ for *T* = 0.29 K. Data representing the ν = 0 insulating state are obtained at *V*_G_ = 0.32 V and Δ*n* = *C*_G_(*V*_G_ – 0.32)/*e*. The bottom row (b), (d), (f), and (h) show the *T* dependence at ν = 0. (a, b) Current *I*_DS_ as a function of source–drain bias across the graphene device *V*_DS_. (c, d) Log-lin plots of *I*_DS_–*V*_DS_. (e, f) ln(*I*_DS_/*V*_DS_^2^) as a function of ln(1/*V*_DS_). Schematic diagrams in (e) represent the barrier shape in different bias regimes. (g, h) Fowler-Nordheim plots of ln(*I*_DS_/*V*_DS_^2^) as a function of 1/*V*_DS_. The vertical dotted line labeled *V*_t_ in (g) corresponds to the inflection point voltage for ν = 0 data, marking a transition between Fowler-Nordheim and direct tunneling. The diagonal dotted line shows a linear fit at high bias for Fowler-Nordheim tunneling. Data at positive *V*_DS_ are shown in (c)–(h). Negative *V*_DS_ shows similar results.

Figure 4a shows the DC current *I*_DS_ as a function of *V*_DS_ for select *V*_G_ at *T* = 0.29 K. The current is obtained by integrating the measured differential conductance *G* = d*I*_DS_/d*V*_B_ over the total DC bias applied to the circuit *V*_B_. The *I*_DS_ remains near zero over the widest range of |*V*_DS_| ≲ 3 mV at ν = 0, an energy scale similar to *E*_a_. Figure 4b shows the temperature dependence for the ν = 0 insulator. Above *T* = 2 K, data become much more linear, as transport becomes thermally activated. The *I*_DS_/*V*_DS_ characteristics are shown on a log-lin scale in Figure 4c,d, for different *n* at *T* = 0.29 K and different *T* at ν = 0, respectively. Positive *V*_DS_ data are plotted in Figure 4c–h. Negative *V*_DS_ data show similar results and are shown in Figure S5. The current changes by nearly 4 orders of magnitude for Δ*V*_DS_ from 0 to ≈4 mV at *T* = 0.29 K and ν = 0. If the barrier length is on the order of ≈100 nm (see later analysis); this corresponds to an electric field strength of ∼0.04 V μm^–1^. The narrow constriction connects to the Ti/Au contact on one side and the wider graphene area on the other. The similarity of data for positive and negative *V*_DS_, Figures 4 and S5, respectively, suggests that any asymmetry caused by different types of contacts to a tunneling region within the constriction is small.

Data at *T* = 0.29 and 2 K cross at high *V*_DS_, likely caused by uncertainties in the series resistance correction. Therefore, only trends at separate *T* will be considered. In this measurement, *R*_S_ depends on *T* and total bias *V*_B_ applied to the circuit. The *V*_B_ dependence of *R*_S_ arises from a gating effect where the bias across the multiplexer changes the Fermi energy in the GaAs 2DEG relative to the addressing gates. The resulting change in resistance manifests as an approximately linear background with DC bias voltage, dependent on *T*, and is corrected for as described in the Supporting Information. The accumulation of estimates makes comparing data between different *T*’s less reliable when *V*_DS_ is the highest and the graphene resistance has become small. It is straightforward to remove the *R*_S_ dependence on DC bias with changes to the multiplexer operation during the experiment by maintaining a fixed potential difference between addressing gates and the 2DEG.^45^ This will allow easier characterization of *I*–*V* curves in the future.

Tunneling mechanisms are investigated by analysis of the current–bias voltage characteristics using eqs 3 and 4.^21−24^Equation 3 describes direct tunneling through a trapezoidal barrier at low bias
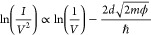
3where ϕ is the barrier height, *m* is the effective mass, and *d* is the length of the tunnel barrier. Plots of ln(*I*/*V*^2^) as a function of ln(1/*V*) show a linear relationship in this regime. At high bias (*V* > ϕ), the barrier becomes triangular and transport can be described using
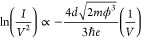
4known as Fowler-Nordheim (FN) tunneling or field emission. Plots of ln(*I*/*V*^2^) as a function of 1/*V* show a linear relationship with a negative gradient. Figure 4e shows ln(*I*_DS_/*V*_DS_^2^) as a function of ln(1/*V*_DS_) at different *V*_G_’s for *T* = 0.29 K. A linear trend is observed at low *V*_DS_, consistent with direct tunneling. As *V*_DS_ increases, an inflection point suggests a transition between tunneling regimes and a changing barrier shape between trapezoidal and triangular. The schematic diagrams indicate the barrier shape at different *V*_DS_’s. A zoom-in of the high *V*_DS_ regime is shown in Figure 4g on a ln(*I*_DS_/*V*_DS_^2^) *vs* 1/*V*_DS_ scale. The vertical dotted line corresponds to the inflection point voltage (*V*_t_) for the ν = 0 state. The negative gradient at high bias is consistent with FN tunneling. The bias energy at the inflection point *eV*_t_ ≈ 1.8 meV is lower than the barrier height *E*_a_ from Fermi–Dirac fitting. Other studies have also observed *eV*_t_ to be smaller than the estimated barrier height,^21^ attributed in part to the approximation of the barrier being simple trapezoidal/triangular shapes instead of being fully treated.

The *T* dependence of the ν = 0 state is shown in Figure 4f,h. Results at *T* = 0.29 and 2 K are consistent with a transition between direct tunneling and FN tunneling. No inflection point occurs at *T* ≥ 5 K, consistent with the *T* at which transport has become thermally activated (*i.e.*, the *G* in Figures 2c and 3c does not change significantly with *T* until *T* > 2 K). Activated carriers are able to overcome the barrier leading to thermionic emission.^22,23^

Equation 4 is the linearized form^21^ of  from which the slope in the FN regime is given by . A linear fit to ν = 0 data at high bias in Figure 4g is shown by a dotted line, which gives barrier length *d* on the order of ≈100 nm. The effective mass is estimated as^46^*m* = *E*/*v*_F_^2^ = (*h*^2^*n*/4π*v*_F_^2^)^(1/2)^ using *v*_F_ = 1 × 10^6^ m s^–1^ and *n* = *n*_0_ (the residual carrier density), since data are obtained at charge neutrality. Barrier height ϕ = *E*_a_ = 2.8 meV is used, provided by the Fermi–Dirac fitting of *V*_DS_ = 0 mV data in Figure 3c. The barrier length is consistent with the estimated Fermi wavelength  at *n* = *n*_0_. The barrier length of ≈100 nm is only given as an approximate estimate, since within the fitting range, the data are not yet truly linear, and it may be that *V*_DS_ is not yet sufficiently high. Further deviations in linearity can occur due to *T* and image effects^20,21,47^ that are not included in eq 4. The equation also assumes a simple triangular barrier shape and is an approximation in the high bias and zero *T* limit. These factors can contribute to uncertainties in the estimate of *d*. Additionally, uncertainties in *R*_S_ at high *V*_DS_ may also cause deviations from linearity. The transition between Fowler-Nordheim and direct tunneling has not previously been reported in this insulating regime, and to our knowledge, detailed *I*–*V* measurements and analysis including temperature dependence have not hitherto been performed. Where *I*–*V* sweeps were carried out at a single *T*, the nonlinear regime indicated an energy scale an order of magnitude larger than the activation energy, suggesting charge transport may occur over several connected insulating areas.^10^ In contrast, our results indicate energy scales similar to *E*_a_, perhaps suggesting an individual insulating region.

An aspect that distinguishes this work is the use of bottom contacts. These have potential to induce strain in graphene next to the contact edge, creating the possibility for the physical origin of these effects to lie in the combination of narrow constriction and strain, where strain from metal contacts can affect device properties.^48^ The high quality region may also indicate cleaner interfaces and low defect density within the narrow constriction, and strict control of the interface quality may be necessary for high yields. We do not observe symmetry breaking and ν = ± 1 states as have been reported in samples with much higher mobilities^5,49^ or measured at much higher magnetic fields.^5^ Similar to refs (2, 3, and 50), this may be attributed to comparatively higher disorder in our devices or insufficiently high *B*. For comparison, devices hosting the high *B* insulating state with very similar mobilities to ours, refs (2 and 3), did not detect ν = ± 1 states for *B* up to 30 and 18 T, respectively. It is also likely that the amount of residual carrier density or disorder in our device precludes the existence of low *B* insulating transitions that appear within the global phase diagram for 2D systems in a magnetic field,^51^ where increasing density can reduce the critical field of the transition to *B* = 0 T.^52^ Instead, our results appear to be consistent with the phase diagram in ref (3).

The insulating state is often observed as *B* is increased,^1−4,10,11,53^ despite the fact that the ground state at ν = 0 is predicted to be a quantum Hall ferromagnet with counter-propagating edge modes, where each edge section contributes *R* = *h*/*e*^2^ and total *R* is given by the combination of these edge sections.^13,14,54−56^ It has recently been shown that quantum Hall ferromagnetism can exist in devices engineered to screen the Coulomb interaction, whereas the insulating ν = 0 state is present in devices where this is not the case.^14^ This was attributed to the indirect modification of lattice–scale electron–electron interactions, which determine the ground state and can lead to either ferromagnetic or insulating phases.^57^

**Figure 5:**
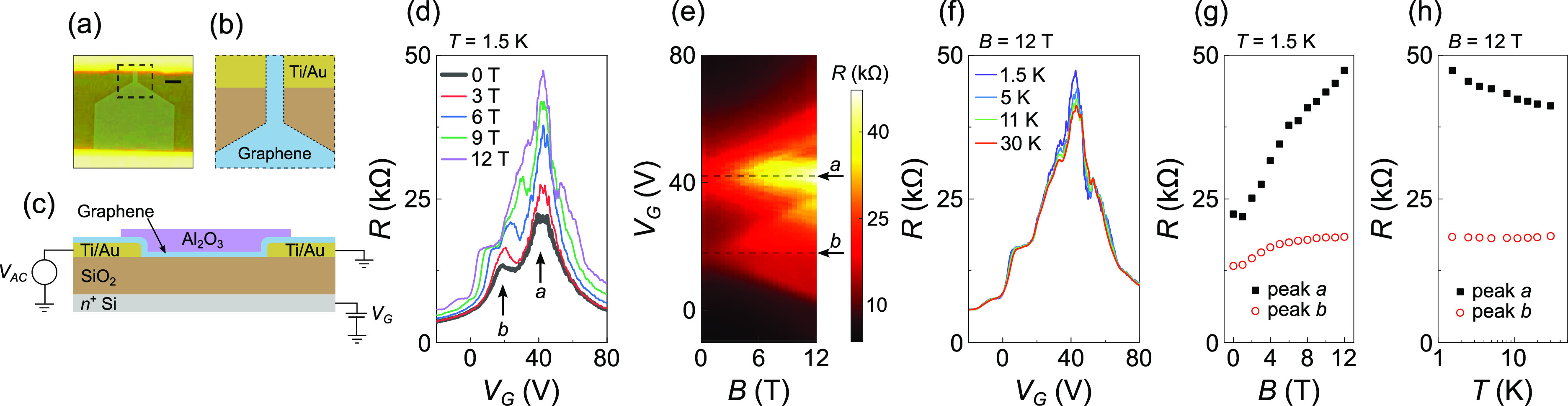
(a) Optical micrograph prior to Al_2_O_3_ deposition with false color graphene (blue) and source–drain contacts (yellow) illustrating the device geometry. The scale bar is 2 μm long. (b) Top down schematic of the lithographic pattern defining the constriction [dashed black box outlined in (a)]; colors are the same as (c). (c) Schematic cross section from source to drain. (d) Magnetic field dependence of transfer characteristics at *T* = 1.5 K. (e) Colormap of *R* as a function of *V*_G_ and *B*. Horizontal dashed lines and arrows indicate charge neutrality points for peaks *a* and *b* as labeled. (f) Temperature dependence of transfer characteristics at *B* = 12 T. (g) Maximum *R* of peak *a* and peak *b* resistance measured at *V*_G_ = 18 V as a function of *B* at *T* = 1.5 K. (h) Resistance *R* as a function of  at *B* = 12 T for peaks *a* and *b*.

The observation of the insulating state, previously requiring the electronic quality given by exfoliation and encapsulation methods, in unencapsulated and wet transferred CVD graphene may be pertinent for the development of graphene-based devices to investigate physics phenomena due to the scalability and relative simplicity of these methods. To address this potential, the role of constriction width and possible strain at the contact edges should be investigated, since here, the behavior appears in a device with a narrow constriction and back contacts. Although the constriction is created unintentionally in the initial device, it is straightforward to determinately recreate with standard lithographic techniques. We deliberately create a similar geometry using electron–beam lithography and oxygen reactive ion etching to define a 10 μm-wide graphene channel with a 600 nm wide constriction next to one contact electrode in monolayer CVD graphene transferred on a doped Si wafer. The device layout is shown in Figure 5a–c, and fabrication and measurement details are given in the Supporting Information. An ∼50 nm Al_2_O_3_ encapsulating layer is added by atomic layer deposition (ALD) after patterning,^58^ and all data presented includes contact resistance.

Figure 5d shows *R* as a function of *V*_G_ at *B* = 0, 3, 6, 9, and 12 T and *T* = 1.5 K. Two clear resistance peaks are visible at *B* = 0 T (bold trace), labeled *a* and *b*. The colormap in Figure 5e reveals their evolution into two sets of Landau levels with charge neutrality points *V*_CNP,*a*_ = ∼42 V and *V*_CNP,*b*_ = ∼18 V for *a* and *b*, respectively, marked by the horizontal dashed lines and arrows. When one considers the graphene channel as two resistive components in series with distinct aspect ratios representing the constriction and main channel, there are indications that the behavior of peak *a* (peak *b*) is consistent with arising from the constriction (wide region). Specifically, the higher *R* of peak *a* at *B* = 0 T is consistent with a larger *L*/*W* ratio, which occurs in constriction, as supported by fitting the peaks separately using eq 1, shown in the Supporting Information. Additionally, nanopatterning graphene (the use of PMMA and planar etching) has been shown to lead to p-doping, which may explain the positive shift of *V*_CNP_ for peak *a* due to the enhanced impact of these doped etched edges on transport through the constriction relative to the wide section. Dual peak behavior is not observed in the unintentional constriction, possibly due to the tearing process resulting in cleaner constriction edges and reduced contamination such that there is no difference in *V*_CNP_ for graphene in the constriction and elsewhere in the device. We speculate that removing the larger graphene area, *i.e.*, fabricating only narrow graphene channels between micron-spaced source–drain electrodes, may remove the secondary Dirac peak.

Figure 5g shows *R* at ν = 0 for peaks *a* and *b* as a function of *B* at *T* = 1.5 K. The resistance of peak *a* rises with *B*, whereas *b* plateaus at ∼18.4 kΩ. The *T* dependence of peaks *a* and *b* at *B* = 12 T is shown in Figure 5h. Peak *a* shows insulating behavior, whereas peak *b* is relatively unchanged in comparison. The behavior of peak *a* is similar to the device with the unintentional constriction in terms of increasing *R* with *B* and insulating *T* dependence. The magnitude of the change in resistance for the insulating behavior is reduced compared to the unintentional constriction, which can be attributed to a combination of the higher base temperature (1.5 K *vs* 300 mK) and increased disorder and doping in the etched edges.^27,28^ It is expected that devices with higher disorder will show a later onset of the insulating state such that *R* is lower at the same *B* and *T*.^1,11,53^ For comparison, Figure 2b shows *R* = ∼860 kΩ at *B* = 10 T and *T* = 2 K. We speculate that less disorder is present in the unintentional constriction since it forms as a result of tearing,^25,26^ such that the edges are never exposed to plasma etching. However, the trends of *R* with *T* and *B* in both devices are the same, and fitting data from Figure 5h using eq 2 estimates an energy gap *E*_a_ of ≈0.9 meV at *B* = 12 T, compared to *E*_a_ of ∼2.4–2.8 meV for the unintentional constriction.

We note that the qualitative difference in the *B* and *T* dependence of peaks *a* and *b* suggests behavior that is inherent to each charge neutrality point, and the effect of overlapping Landau levels is small. If this were not the case, both peaks might be expected to show the same behavior, since peak *b* overlaps *a* and *vice versa*. The strong *T* dependence at peak *a* in Figure 5f compared to the little discernible change around peak *b* and for *V*_G_ < 18 V (*i.e.*, in the region of Landau levels from peak *b*) also suggests the *T* dependence around *V*_G_ = ∼42 V (*V*_CNP,*a*_) can be attributed to the ν = 0 state of peak *a* rather than overlapping Landau levels.

These data provide evidence that the insulating state at ν = 0 can be reproduced in deliberately fabricated CVD graphene devices and that the unintentional constriction may be benefiting from higher electronic quality due to the presumably torn edges. This suggests the importance of lithographic patterning processes that minimize edge disorder and contamination, such as using *h*-BN masks,^27,28^ when creating deterministically defined constrictions, and may imply the possibility for systems to investigate exotic physics using relatively simple and scalable fabrication methods with the implementation of such techniques. Our results highlight the use of multiplexing in driving device development, since by studying the device conditions leading to the initial observation of the insulating state, we are able to reproduce it deterministically and identify key factors for its existence. Additionally, the multiplexing platform combined with scalable fabrication such as wet transfer techniques of CVD material has demonstrated a convenient means by which devices can be identified for fundamental physics research or with functional properties.

Future expansion of the multiplexing tool to achieve four-probe capabilities and incorporate *h*-BN substrates or *h*-BN encapsulation presents intriguing possibilities to explore other diverse phenomena, including those in graphene devices on patterned *h*-BN.^59^ The *N* = 0 Landau level in hybrid graphene systems has also shown intriguing behavior and could be investigated.^60,61^ Expansion to other nanomaterials is straightforward since nanowires, exfoliated 2D materials, and CVD-grown 2D materials can be transferred to the multiplexer using compatible fabrication methods.^16^ The onset of the high resistance state studied here in graphene has been shown to occur at lower magnetic fields in devices with higher mobility and lower disorder.^1,11,53^ The reduction of the magnetic field required may bring CVD-grown graphene devices into the realm of giant magnetoresistance devices and magnetic switches^62^ and motivate the development of graphene-based magnetic field detection or magnetic texture mapping sensors with cryogenic readout.

## Conclusions

These results show the existence of a magnetic field-induced insulating state in monolayer CVD graphene with resistance that diverges with *B* at charge neutrality. This state is usually associated with very high quality devices made using exfoliated graphene flakes and suspended, encapsulated, or fabricated on extremely smooth substrates such as hexagonal boron nitride. The device studied here uses CVD-grown graphene that is unencapsulated and fabricated on an Al_2_O_3_ substrate created by atomic layer deposition. The insulating state shows activated behavior with an energy gap that increases with *B*. DC source–drain bias measurements reveal a transition between direct tunneling and Fowler-Nordheim tunneling at *T* = 0.29 and 2 K as bias increases. The results support the existence of a gapped region with a length on the order of ≈100 nm in an ≈440 nm-wide constriction created within a larger area of graphene. This work demonstrates the opportunistic study of properties serendipitously created in a device within a multiplexed array. High-throughput testing *via* the multiplexing platform increases the opportunity to identify such devices with unusual properties and study them and is compatible with different nanoelectronic devices including 2D materials and semiconductor nanowires, measured from cryogenic to room temperatures.^16^

## Methods

These methods concern the primary device on the multiplexing platform. Methods relating to the second device are provided in the Supporting Information. The multiplexer is fabricated in a GaAs high electron mobility transistor (HEMT) in which a 2DEG forms 90 nm below the surface (Cavendish wafer V832). The mean sheet density and electron mobility are *n*_GaAs_ = 1.53 × 10^11^ cm^–2^ and μ_GaAs_ = 8.17 × 10^5^ cm^2^ V^–1^s^–1^, respectively, measured on a separate Hall bar device. A conducting path is defined within the 2DEG from one input to 16 outputs, and individual outputs are chosen by surface addressing gates. Multiplexer layout, fabrication, and operation details are described in ref (16), although the device measured here is different from that in ref (16). Source–drain contacts (Ti/Au) are fabricated on the multiplexer before transferring a monolayer graphene film grown by chemical vapor deposition onto Cu. The graphene is coated with poly(methyl methacrylate) (PMMA), and the Cu is etched using a 1.2% solution of ammonium persulfate etchant (NH_4_)_2_S_2_O_8_. The PMMA–graphene stack is transferred to the multiplexer after rinsing in a DI water bath and dried in air overnight before baking at 125 °C for 30 min and soaking in acetone to remove the PMMA. Graphene channels (10 μm wide) are defined between source–drain contacts (10 μm separation) by photolithography and oxygen reactive ion etching. For the device measured here, Raman measurements show a large 2D to G peak ratio across the entirety of the graphene, consistent with a single layer, shown in Figure S6. The two-terminal differential conductance is measured using 77 Hz AC voltage, *V*_AC_ = 400 μV (rms), applied to the multiplexer and graphene in series. At *B* = 10 T, the maximum power dissipated in the graphene and energy loss rate per carrier are estimated to be <0.2 pW and <1.5 × 10^–18^ W, respectively, which suggests a carrier temperature of <0.6 K, as discussed in the Supporting Information.

## Supporting Information Available

Series resistance, energy gaps estimated using an Arrhenius equation, carrier mobility estimated using the quantum Hall effect, DC source–drain bias across the graphene, effective gating of the graphene due to source–drain bias, DC current–voltage characteristics for negative *V*_DS_, energy loss rates and carrier temperature, Raman spectroscopy, and methods for the deliberately defined constriction (PDF)

## Notes

The data set for this Article is available at 10.17863/CAM.65353.

## References

[ref1] Checkelsky J. G.; Li L.; Ong N. P. Zero-Energy State in Graphene in a High Magnetic Field. Phys. Rev. Lett. 2008, 100, 206801 10.1103/PhysRevLett.100.206801. 18518564

[ref2] Giesbers A. J. M.; Ponomarenko L. A.; Novoselov K. S.; Geim A. K.; Katsnelson M. I.; Maan J. C.; Zeitler U. Gap Opening in the Zeroth Landau Level of Graphene. Phys. Rev. B 2009, 80, 201403 10.1103/PhysRevB.80.201403.

[ref3] Zhang L.; Zhang Y.; Khodas M.; Valla T.; Zaliznyak I. A. Metal to Insulator Transition on the *N* = 0 Landau Level in Graphene. Phys. Rev. Lett. 2010, 105, 046804 10.1103/PhysRevLett.105.046804. 20867875

[ref4] Zhang L.; Camacho J.; Cao H.; Chen Y. P.; Khodas M.; Kharzeev D. E.; Tsvelik A. M.; Valla T.; Zaliznyak I. A. Breakdown of the *N* = 0 Quantum Hall State in Graphene: Two Insulating Regimes. Phys. Rev. B 2009, 80, 241412 10.1103/PhysRevB.80.241412.

[ref5] Zhang Y.; Jiang Z.; Small J. P.; Purewal M. S.; Tan Y.-W.; Fazlollahi M.; Chudow J. D.; Jaszczak J. A.; Stormer H. L.; Kim P. Landau-Level Splitting in Graphene in High Magnetic Fields. Phys. Rev. Lett. 2006, 96, 136806 10.1103/PhysRevLett.96.136806. 16712020

[ref6] Amado M.; Diez E.; López-Romero D.; Rossella F.; Caridad J. M.; Dionigi F.; Bellani V.; Maude D. K. Plateau–Insulator Transition in Graphene. New J. Phys. 2010, 12, 053004 10.1088/1367-2630/12/5/053004.

[ref7] Banszerus L.; Schmitz M.; Engels S.; Goldsche M.; Watanabe K.; Taniguchi T.; Beschoten B.; Stampfer C. Ballistic Transport Exceeding 28 μm in CVD Grown Graphene. Nano Lett. 2016, 16, 1387–1391. 10.1021/acs.nanolett.5b04840. 26761190

[ref8] De Fazio D.; Purdie D. G.; Ott A. K.; Braeuninger-Weimer P.; Khodkov T.; Goossens S.; Taniguchi T.; Watanabe K.; Livreri P.; Koppens F. H. L.; Hofmann S.; Goykhman I.; Ferrari A. C.; Lombardo A. High-Mobility, Wet-Transferred Graphene Grown by Chemical Vapor Deposition. ACS Nano 2019, 13, 8926–8935. 10.1021/acsnano.9b02621. 31322332

[ref9] Pezzini S.; Mišeikis V.; Pace S.; Rossella F.; Watanabe K.; Taniguchi T.; Coletti C. High-Quality Electrical Transport Using Scalable CVD Graphene. 2D Materials 2020, 7, 041003 10.1088/2053-1583/aba645.

[ref10] Bolotin K. I.; Ghahari F.; Shulman M. D.; Stormer H. L.; Kim P. Observation of the Fractional Quantum Hall Effect in Graphene. Nature 2009, 462, 196–199. 10.1038/nature08582. 19881489

[ref11] Du X.; Skachko I.; Duerr F.; Luican A.; Andrei E. Y. Fractional Quantum Hall Effect and Insulating Phase of Dirac Electrons in Graphene. Nature 2009, 462, 192–195. 10.1038/nature08522. 19829294

[ref12] Young A. F.; Sanchez-Yamagishi J. D.; Hunt B.; Choi S. H.; Watanabe K.; Taniguchi T.; Ashoori R. C.; Jarillo-Herrero P. Tunable Symmetry Breaking and Helical Edge Transport in a Graphene Quantum Spin Hall State. Nature 2014, 505, 528–532. 10.1038/nature12800. 24362569

[ref13] Young A. F.; Dean C. R.; Wang L.; Ren H.; Cadden-Zimansky P.; Watanabe K.; Taniguchi T.; Hone J.; Shepard K. L.; Kim P. Spin and Valley Quantum Hall Ferromagnetism in Graphene. Nat. Phys. 2012, 8, 550–556. 10.1038/nphys2307.

[ref14] Veyrat L.; Déprez C.; Coissard A.; Li X.; Gay F.; Watanabe K.; Taniguchi T.; Han Z.; Piot B. A.; Sellier H.; Sacépé B. Helical Quantum Hall Phase in Graphene on SrTiO_3_. Science 2020, 367, 781 10.1126/science.aax8201. 32054761

[ref15] Chiappini F.; Wiedmann S.; Novoselov K.; Mishchenko A.; Geim A. K.; Maan J. C.; Zeitler U. Lifting of the Landau Level Degeneracy in Graphene Devices in a Tilted Magnetic Field. Phys. Rev. B 2015, 92, 201412 10.1103/PhysRevB.92.201412.

[ref16] Smith L. W.; Batey J. O.; Alexander-Webber J. A.; Fan Y.; Hsieh Y.-C.; Fung S.-J.; Jevtics D.; Robertson J.; Guilhabert B. J. E.; Strain M. J.; Dawson M. D.; Hurtado A.; Griffiths J. P.; Beere H. E.; Jagadish C.; Burton O. J.; Hofmann S.; Chen T.-M.; Ritchie D. A.; Kelly M.; et al. High-Throughput Electrical Characterization of Nanomaterials from Room to Cryogenic Temperatures. ACS Nano 2020, 14, 15293–15305. 10.1021/acsnano.0c05622. 33104341

[ref17] Pauka S.; Das K.; Hornibrook J.; Gardner G.; Manfra M.; Cassidy M.; Reilly D. Characterizing Quantum Devices at Scale with Custom Cryo-CMOS. Phys. Rev. Appl. 2020, 13, 054072 10.1103/PhysRevApplied.13.054072.

[ref18] Paquelet Wuetz B.; Bavdaz P. L.; Yeoh L. A.; Schouten R.; van der Does H.; Tiggelman M.; Sabbagh D.; Sammak A.; Almudever C. G.; Sebastiano F.; Clarke J. S.; Veldhorst M.; Scappucci G. Multiplexed Quantum Transport Using Commercial Off-the-Shelf CMOS at Sub-Kelvin Temperatures. npj Quantum Inf. 2020, 6, 43 10.1038/s41534-020-0274-4.

[ref19] Al-Taie H.; Smith L. W.; Xu B.; See P.; Griffiths J. P.; Beere H. E.; Jones G. A. C.; Ritchie D. A.; Kelly M. J.; Smith C. G. Cryogenic On-Chip Multiplexer for the Study of Quantum Transport in 256 Split-Gate Devices. Appl. Phys. Lett. 2013, 102, 243102 10.1063/1.4811376.

[ref20] Lenzlinger M.; Snow E. H. Fowler-Nordheim Tunneling into Thermally Grown SiO_2_. J. Appl. Phys. 1969, 40, 278–283. 10.1063/1.1657043.

[ref21] Beebe J. M.; Kim B.; Gadzuk J. W.; Daniel Frisbie C.; Kushmerick J. G. Transition from Direct Tunneling to Field Emission in Metal-Molecule-Metal Junctions. Phys. Rev. Lett. 2006, 97, 026801 10.1103/PhysRevLett.97.026801. 16907471

[ref22] Sarker B. K.; Khondaker S. I. Thermionic Emission and Tunneling at Carbon Nanotube–Organic Semiconductor Interface. ACS Nano 2012, 6, 4993–4999. 10.1021/nn300544v. 22559008

[ref23] Pramod K.; Gangineni R. B. Rectifying Electronic Transport and the Role of Fowler-Nordheim Tunneling in Ag/PVDF/Au Capacitor Structures. Curr. Appl. Phys. 2017, 17, 1469–1475. 10.1016/j.cap.2017.08.012.

[ref24] Lee T.; Wang W.; Reed M. A. Mechanism of Electron Conduction in Self-Assembled Alkanethiol Monolayer Devices. Ann. N.Y. Acad. Sci. 2003, 1006, 21–35. 10.1196/annals.1292.001. 14976007

[ref25] Kim K.; Artyukhov V. I.; Regan W.; Liu Y.; Crommie M. F.; Yakobson B. I.; Zettl A. Ripping Graphene: Preferred Directions. Nano Lett. 2012, 12, 293–297. 10.1021/nl203547z. 22149252

[ref26] Kim K.; Coh S.; Kisielowski C.; Crommie M. F.; Louie S. G.; Cohen M. L.; Zettl A. Atomically Perfect Torn Graphene Edges and Their Reversible Reconstruction. Nat. Commun. 2013, 4, 2723 10.1038/ncomms3723. 24177166

[ref27] Danielsen D. R.; Lyksborg-Andersen A.; Nielsen K. E. S.; Jessen B. S.; Booth T. J.; Doan M.-H.; Zhou Y.; Bøggild P.; Gammelgaard L. Super-Resolution Nanolithography of Two-Dimensional Materials by Anisotropic Etching. ACS Appl. Mater. Interfaces 2021, 13, 41886–41894. 10.1021/acsami.1c09923. 34431654

[ref28] Jessen B. S.; Gammelgaard L.; Thomsen M. R.; Mackenzie D. M. A.; Thomsen J. D.; Caridad J. M.; Duegaard E.; Watanabe K.; Taniguchi T.; Booth T. J.; Pedersen T. G.; Jauho A.-P.; Bøggild P. Lithographic Band Structure Engineering of Graphene. Nat. Nanotechnol. 2019, 14, 340–346. 10.1038/s41565-019-0376-3. 30778216

[ref29] Smith L. W.; Al-Taie H.; Lesage A. A. J.; Sfigakis F.; See P.; Griffiths J. P.; Beere H. E.; Jones G. A. C.; Ritchie D. A.; Hamilton A. R.; Kelly M. J.; Smith C. G. Dependence of the 0.7 Anomaly on the Curvature of the Potential Barrier in Quantum Wires. Phys. Rev. B 2015, 91, 235402 10.1103/PhysRevB.91.235402.

[ref30] Smith L. W.; Al-Taie H.; Lesage A. A. J.; Thomas K. J.; Sfigakis F.; See P.; Griffiths J. P.; Farrer I.; Jones G. A. C.; Ritchie D. A.; Kelly M. J.; Smith C. G. Effect of Split Gate Size on the Electrostatic Potential and 0.7 Anomaly Within Quantum Wires on a Modulation-Doped GaAs/AlGaAs Heterostructure. Phys. Rev. Appl. 2016, 5, 044015 10.1103/PhysRevApplied.5.044015.

[ref31] Smith L. W.; Al-Taie H.; Sfigakis F.; See P.; Lesage A. A. J.; Xu B.; Griffiths J. P.; Beere H. E.; Jones G. A. C.; Ritchie D. A.; Kelly M. J.; Smith C. G. Statistical Study of Conductance Properties in One-Dimensional Quantum Wires Focusing on the 0.7 Anomaly. Phys. Rev. B 2014, 90, 045426 10.1103/PhysRevB.90.045426.

[ref32] Puddy R. K.; Smith L. W.; Al-Taie H.; Chong C. H.; Farrer I.; Griffiths J. P.; Ritchie D. A.; Kelly M. J.; Pepper M.; Smith C. G. Multiplexed Charge-Locking Device for Large Arrays of Quantum Devices. Appl. Phys. Lett. 2015, 107, 143501 10.1063/1.4932012.

[ref33] Lesage A. A. J.; Smith L. W.; Al-Taie H.; See P.; Griffiths J. P.; Farrer I.; Jones G. A. C.; Ritchie D. A.; Kelly M. J.; Smith C. G. Assisted Extraction of the Energy Level Spacings and Lever Arms in Direct Current Bias Measurements of One-Dimensional Quantum Wires, Using an Image Recognition Routine. J. Appl. Phys. 2015, 117, 015704 10.1063/1.4905484.

[ref34] Suk J. W.; Kitt A.; Magnuson C. W.; Hao Y.; Ahmed S.; An J.; Swan A. K.; Goldberg B. B.; Ruoff R. S. Transfer of CVD-Grown Monolayer Graphene onto Arbitrary Substrates. ACS Nano 2011, 5, 6916–6924. 10.1021/nn201207c. 21894965

[ref35] Kim S.; Nah J.; Jo I.; Shahrjerdi D.; Colombo L.; Yao Z.; Tutuc E.; Banerjee S. K. Realization of a High Mobility Dual-Gated Graphene Field-Effect Transistor With Al_2_O_3_ Dielectric. Appl. Phys. Lett. 2009, 94, 062107 10.1063/1.3077021.

[ref36] Zhong H.; Zhang Z.; Xu H.; Qiu C.; Peng L.-M. Comparison of Mobility Extraction Methods Based on Field-Effect Measurements for Graphene. AIP Advances 2015, 5, 057136 10.1063/1.4921400.

[ref37] Novoselov K. S.; Geim A. K.; Morozov S. V.; Jiang D.; Zhang Y.; Dubonos S. V.; Grigorieva I. V.; Firsov A. A. Electric Field Effect in Atomically Thin Carbon Films. Science 2004, 306, 666–669. 10.1126/science.1102896. 15499015

[ref38] Zhang Y.; Tan Y.-W.; Stormer H. L.; Kim P. Experimental Observation of the Quantum Hall Effect and Berry’s Phase in Graphene. Nature 2005, 438, 201–204. 10.1038/nature04235. 16281031

[ref39] Novoselov K. S.; Geim A. K.; Morozov S. V.; Jiang D.; Katsnelson M. I.; Grigorieva I. V.; Dubonos S. V.; Firsov A. A. Two-Dimensional Gas of Massless Dirac Fermions in Graphene. Nature 2005, 438, 197–200. 10.1038/nature04233. 16281030

[ref40] Foa Torres L. E. F.; Roche S.; Charlier J.-C. Introduction to Graphene-Based Nanomaterials: From Electronic Structure to Quantum Transport; Cambridge University Press: Cambridge, U.K., 2014.

[ref41] Kurganova E. V.; Giesbers A. J. M.; Gorbachev R. V.; Geim A. K.; Novoselov K. S.; Maan J. C.; Zeitler U. Quantum Hall Activation Gaps in Bilayer Graphene. Solid State Commun. 2010, 150, 2209–2211. 10.1016/j.ssc.2010.09.042.

[ref42] Kurganova E. V.; van Elferen H. J.; McCollam A.; Ponomarenko L. A.; Novoselov K. S.; Veligura A.; van Wees B. J.; Maan J. C.; Zeitler U. Spin Splitting in Graphene Studied by Means of Tilted Magnetic-Field Experiments. Phys. Rev. B 2011, 84, 121407 10.1103/PhysRevB.84.121407.

[ref43] Hwang E. H.; Das Sarma S. Single-Particle Relaxation Time *Versus* Transport Scattering Time in a Two-Dimensional Graphene Layer. Phys. Rev. B 2008, 77, 195412 10.1103/PhysRevB.77.195412.

[ref44] Baker A. M. R.; Alexander-Webber J. A.; Altebaeumer T.; McMullan S. D.; Janssen T. J. B. M.; Tzalenchuk A.; Lara-Avila S.; Kubatkin S.; Yakimova R.; Lin C.-T.; Li L.-J.; Nicholas R. J. Energy Loss Rates of Hot Dirac Fermions in Epitaxial, Exfoliated, and CVD Graphene. Phys. Rev. B 2013, 87, 045414 10.1103/PhysRevB.87.045414.

[ref45] Yi T. Progress Towards GaAs Multiplexed Single-Electron Pump Arrays, Ph.D. thesis, University of Cambridge, 2019.

[ref46] Novoselov K. S.; Jiang D.; Schedin F.; Booth T. J.; Khotkevich V. V.; Morozov S. V.; Geim A. K. Two-Dimensional Atomic Crystals. Proc. Natl. Acad. Sci. U. S. A. 2005, 102, 10451–10453. 10.1073/pnas.0502848102. 16027370 PMC1180777

[ref47] Koehler M.; Hümmelgen I. A. Temperature Dependent Tunnelling Current at Metal/Polymer Interfaces—Potential Barrier Height Determination. Appl. Phys. Lett. 1997, 70, 3254–3256. 10.1063/1.119149.

[ref48] De Sanctis A.; Mehew J. D.; Alkhalifa S.; Withers F.; Craciun M. F.; Russo S. Strain-Engineering of Twist-Angle in Graphene/hBN Superlattice Devices. Nano Lett. 2018, 18, 7919–7926. 10.1021/acs.nanolett.8b03854. 30474986

[ref49] Dean C. R.; Young A. F.; Meric I.; Lee C.; Wang L.; Sorgenfrei S.; Watanabe K.; Taniguchi T.; Kim P.; Shepard K. L.; Hone J. Boron Nitride Substrates for High-Quality Graphene Electronics. Nat. Nanotechnol. 2010, 5, 722–726. 10.1038/nnano.2010.172. 20729834

[ref50] Giesbers A. J. M.; Zeitler U.; Katsnelson M. I.; Ponomarenko L. A.; Mohiuddin T. M.; Maan J. C. Quantum-Hall Activation Gaps in Graphene. Phys. Rev. Lett. 2007, 99, 206803 10.1103/PhysRevLett.99.206803. 18233175

[ref51] Kivelson S.; Lee D.-H.; Zhang S.-C. Global Phase Diagram in the Quantum Hall Effect. Phys. Rev. B 1992, 46, 2223–2238. 10.1103/PhysRevB.46.2223. 10003898

[ref52] Hanein Y.; Nenadovic N.; Shahar D.; Shtrikman H.; Yoon J.; Li C. C.; Tsui D. C. Linking Insulator-To-Metal Transitions at Zero and Finite Magnetic Fields. Nature 1999, 400, 735–737. 10.1038/23419.

[ref53] Checkelsky J. G.; Li L.; Ong N. P. Divergent Resistance at the Dirac Point in Graphene: Evidence for a Transition in a High Magnetic Field. Phys. Rev. B 2009, 79, 115434 10.1103/PhysRevB.79.115434.

[ref54] Abanin D. A.; Lee P. A.; Levitov L. S. Spin-Filtered Edge States and Quantum Hall Effect in Graphene. Phys. Rev. Lett. 2006, 96, 176803 10.1103/PhysRevLett.96.176803. 16712323

[ref55] Abanin D. A.; Novoselov K. S.; Zeitler U.; Lee P. A.; Geim A. K.; Levitov L. S. Dissipative Quantum Hall Effect in Graphene near the Dirac Point. Phys. Rev. Lett. 2007, 98, 196806 10.1103/PhysRevLett.98.196806. 17677649

[ref56] Nomura K.; MacDonald A. H. Quantum Hall Ferromagnetism in Graphene. Phys. Rev. Lett. 2006, 96, 256602 10.1103/PhysRevLett.96.256602. 16907331

[ref57] Kharitonov M. Phase Diagram for the ν = 0 Quantum Hall State in Monolayer Graphene. Phys. Rev. B 2012, 85, 155439 10.1103/PhysRevB.85.155439.

[ref58] Alexander-Webber J. A.; Sagade A. A.; Aria A. I.; Van Veldhoven Z. A.; Braeuninger-Weimer P.; Wang R.; Cabrero-Vilatela A.; Martin M.-B.; Sui J.; Connolly M. R.; Hofmann S. Encapsulation of Graphene Transistors and Vertical Device Integration by Interface Engineering with Atomic Layer Deposited Oxide. 2D Mater. 2017, 4, 011008 10.1088/2053-1583/4/1/011008.

[ref59] Ho S.-C.; Chang C.-H.; Hsieh Y.-C.; Lo S.-T.; Huang B.; Vu T.-H.-Y.; Ortix C.; Chen T.-M. Hall Effects in Artificially Corrugated Bilayer Graphene Without Breaking Time-Reversal Symmetry. Nature Electronics 2021, 4, 116–125. 10.1038/s41928-021-00537-5.

[ref60] Cadden-Zimansky P.; Shinn M.; Myers G. T.; Chu Y.; Dalrymple M. J.; Travaglini H. C. Formation of the n = 0 Landau Level in Hybrid Graphene. Journal of Physics Communications 2018, 2, 051001 10.1088/2399-6528/aabe3f.

[ref61] Li G.; Luican A.; Andrei E. Y. Scanning Tunneling Spectroscopy of Graphene on Graphite. Phys. Rev. Lett. 2009, 102, 176804 10.1103/PhysRevLett.102.176804. 19518809

[ref62] Weiss R.; Mattheis R.; Reiss G. Advanced Giant Magnetoresistance Technology for Measurement Applications. Measurement Science and Technology 2013, 24, 082001 10.1088/0957-0233/24/8/082001.

