# Supplementary material for: Giant Magnetoresistance in a Chemical Vapor Deposition Graphene Constriction
Source: ACS Nano. 2022 Feb 3;16(2):2833–42. doi: 10.1021/acsnano.1c09815 (PMC9098165; doi:10.1021/acsnano.1c09815)
Supplement: Supplementary file 1 — nn1c09815_si_001.pdf [file nn1c09815_si_001.pdf]

Supporting Information: Giant Magnetoresistance in a Chemical Vapor Deposition Graphene Constriction

Luke W. Smith,*,†,‡ Jack O. Batey,† Jack A. Alexander-Webber,¶ Yu-Chiang Hsieh,‡ Shin-Jr Fung,‡ Tom Albrow-Owen,¶ Harvey E. Beere,† Oliver J. Burton,¶ Stephan Hofmann,¶ David A. Ritchie,† Michael Kelly,¶,† Tse-Ming Chen,‡ Hannah J. Joyce,¶ and Charles G. Smith†

†*Department of Physics, Cavendish Laboratory, University of Cambridge, Cambridge CB3 0HE, U.K.*

‡*Department of Physics, National Cheng Kung University, Tainan 701, Taiwan*

¶*Electrical Engineering Division, Department of Engineering, University of Cambridge, Cambridge CB3 0FA, U.K.*

E-mail: lukesmith@phys.ncku.edu.tw

Series resistance. Figure S1(a) and (b) show data from Figure 2(a) and (b) in the main article, respectively, without subtracting series resistance R_S. Figure S1(a) shows the measured resistance R as a function of V_G for $B = 7$, 8, 9, and 10 T at $T = 0.29$ K. Figure S1(b) shows the resistance at $V_G = 0.31$ V as a function of B for different T from 0.29 to 25 K, and represents the $\nu = 0$ insulating state.

The series resistance is estimated using $R_S(B,T) = R_P(0, 0.29) + \Delta R_{2DEG}(B,T) + \Delta R_P(0,T)$, where $R_P(0, 0.29)$ is the parasitic resistance of the circuit at $T = 0.29$ K and

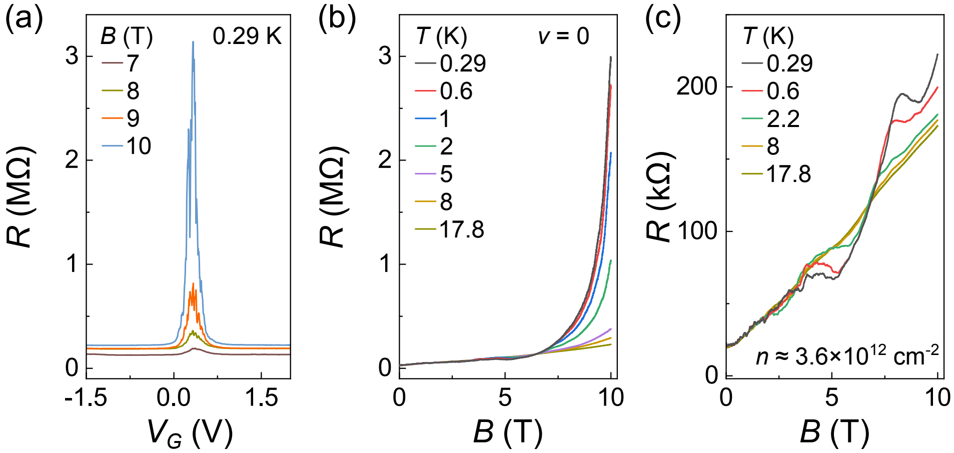

Figure S1: Series resistance. (a and b) Data from Figure 2(a) and (b), main article, before subtracting series resistance R_S. (a) Resistance as a function of back gate voltage V_G for B = 7, 8, 9, and 10 T, at T = 0.29 K. (b) Resistance as a function of magnetic field for different T at the $\nu = 0$ insulating state, measured at V_G = 0.31 V. (c) Resistance as a function of B at high carrier density, for T from 0.29 to 25 K.

$B = 0$ T, $\Delta R_{2DEG}(B,T)$ is the B- and T-dependent change in multiplexer 2DEG resistance, and $\Delta R_P(0,T)$ is the T-dependent change in parasitic resistance at $B = 0$ T. The effect of DC bias on R_S will be considered later. At the magnetic fields used in this work $\Delta R_{2DEG}(B,T)$ is the dominant term, *e.g.*, $\Delta R_{2DEG}(B,T)$ = 200, 177, 159, 157, 156, 154, 154, 153 and 151 kΩ for T = 0.29, 0.6, 2.2, 5, 8, 11.3, 14, 17.8, and 25 K, respectively, at B = 10 T. Values are estimated from the change in R between B = 0 and B = 10 T at V_G = 10 V ($n \approx 3.6 \times 10^{12}$ cm^{-2}), shown in Figure S1(c), in which R is plotted as a function of B at V_G = 10 V for different T. Large $|V_G|$ is used to minimise the graphene resistance, such that the multiplexer resistance dominates. The resistance $R_P(0, 0.29)$ is estimated from fitting[1] transfer characteristics in Figure 1(d), main article at B = 0 T and T = 0.29 K using eq 1, which gives $R_{P,e}$ = 19 kΩ, and includes the zero-field multiplexer 2DEG resistance, Ohmic contacts, and circuit/cryostat wiring.[2] This value is an overestimate[3] since fitting assumes R at high density is only from contacts, without accounting for other effects. The T-dependent $\Delta R_P(0,T)$ is estimated from the change of R with T at B = 0 T and V_G = 10 V. From Figure S1(c) $\Delta R_P(0,T)$ = −0.1, −0.6, −1.1, −1.6, −2.0, −2.3, −2.7, and −3.2 kΩ as T changes from 0.29 K to 0.6, 2.2, 5, 8, 11.3, 14, 17.8 and 25 K, respectively, which

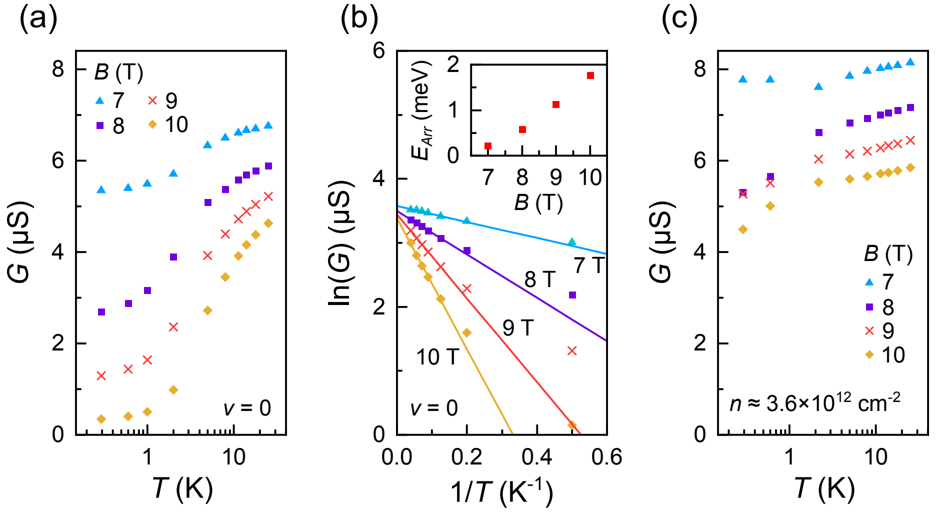

Figure S2: (a) Conductance at $B = 7$, 8, 9, and 10 T as a function of temperature at the $\nu = 0$ insulating state. Data corresponds to Figure 2(c), main article, before subtracting series resistance. (b) Arrhenius plots of G at $\nu = 0$ for fixed magnetic fields $B = 7$, 8, 9, and 10 T. Straight lines show fits using the Arrhenius equation, using data $T \geq 8$ K for fitting. The inset shows activation energy as a function of magnetic field. (c) Conductance at $B = 7$, 8, 9, and 10 T as a function of temperature high carrier density. Series resistance is subtracted in (b) to compare fitting results with Figure 2(c), main article, but not in (a) or (c). The legend is the same for (a)–(c).

corresponds to $\approx 0.05\%$ to $\approx 2\%$ of $\Delta R_{2DEG}(B, T)$ for T from 0.6 to 25 K.

For completeness, Figure S2(a) shows data from Figure 2(c), main article, without subtracting R_S. The conductance of the $\nu = 0$ insulating state at $B = 7$, 8, 9, and 10 T is plotted as a function of T on a lin-log scale. Figure S2(c) shows the corresponding data at high carrier density, $n \approx 3.6 \times 10^{12}$ cm^{-2}, away from the insulating state. Figure S2(a) and (c) are plotted on the same scale to illustrate differences in the data. The conductance G in Figure S2(c) reduces at lower T, whereas G becomes relatively T-independent at low T in Figure S2(a).

Energy gaps estimated using an Arrhenius equation. A Fermi-Dirac function[4,5] is used in the main article to estimate energy gaps E_a, since fitting with an Arrhenius equation[5–13] gives energy gaps within the range of thermal energies k_BT over which measurements are performed ($k_BT \approx 2.2$ meV at $T = 25$ K, where k_B is the Boltzmann constant). Figure

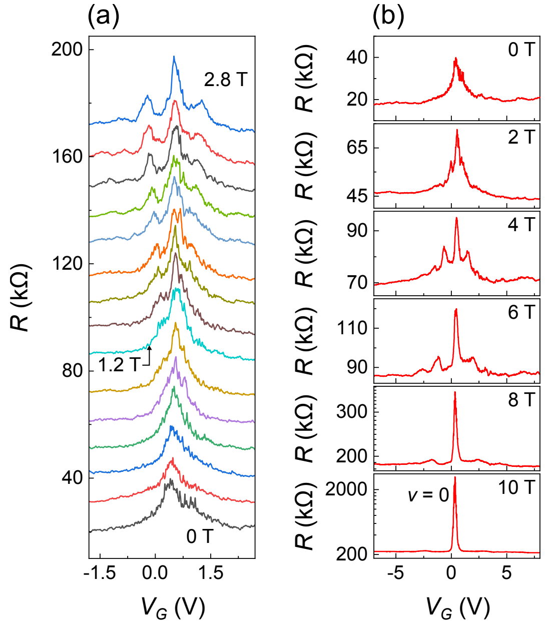

Figure S3: Magnetic field dependent transfer characteristics at $T = 0.29$ K. (a) Resistance R as a function of V_G from $B = 0$ to 2.8 T (bottom-to-top), $\Delta B = 0.2$ T between traces. Traces are offset vertically for clarity. (b) Transfer characteristics at $B = 0$, 2, 4, 6, 8, and 10 T. The vertical axes of $B = 8$ and 10 T panels are plotted on a log scale because of the diverging resistance of the peak at $\nu = 0$ (labeled). Series resistance is not subtracted in (a) or (b).

S2(b) shows an Arrhenius plot of G at $\nu = 0$ for $B = 7$, 8, 9, and 10 T, after subtracting series resistance. Straight lines are fits using the Arrhenius equation $G \propto \exp(-E_{Arr}/2k_BT)$, giving energy gap $E_{Arr} = 0.2$, 0.6, 1.1, and 1.8 meV at $B = 7$, 8, 9, and 10 T, respectively. The fitting is performed using $T \geq 8$ K data. At low T the conductance tends towards T-independent values. The inset shows E_{Arr} as a function of B. A linear fit of $E_{Arr} = \Delta E_Z - \Gamma$ where Zeeman energy $\Delta E_Z = g\mu_B B$ gives an effective Landé g-factor $g \approx 9$ and disorder broadening of Landau levels $\Gamma = 40.5$ K.

Carrier mobility estimated using the quantum Hall effect. For Landau quantization to be observed in magnetotransport measurements,[14] $\mu B \gg 1$ or $\tau\omega_c \gg 1$, where μ is the carrier mobility, τ is the scattering time, and ω_c is the cyclotron frequency. This can be used to estimate μ and τ using the approximations $\mu \approx 1/B$ and $\tau \approx 1/\omega_c$ at the minimum B for which Landau levels are resolved. Figure S3(a) shows transfer characteris-

tics R as a function of V_G from $B = 0$ to 2.8 T (bottom-to-top), with $\Delta B = 0.2$ T between traces. Traces are offset vertically for clarity. Landau levels $N \pm 1$ appear as peaks in R as B increases, and Landau level $N = -1$ first becomes visible as a shoulder in R at $B = 1.2$ T ($B = 1.2$ T data are labeled). This gives $\mu = 1/B \approx 8000$ cm^2 V^{-1}s^{-1} and $\tau = 1/\omega_c \approx 17$ fs, where $\omega_c = v_F\sqrt{2eB/\hbar}$, and Fermi velocity $v_F = 1 \times 10^6$ m s^{-1}. The mean free path $l = v_F\tau \approx 17$ nm. Figure S3(b) shows transfer characteristics for B from 0 to 10 T, at $B = 2$ T intervals. At high B the resistance at charge neutrality becomes much larger than resistance peaks associated with higher index Landau levels, therefore R is plotted on a log scale for $B = 8$ and 10 T.

DC source–drain bias across the graphene. The DC source–drain voltage across the graphene V_{DS} is given by $V_{DS} = V_B - I_{DS}R_S - I_{DS}\Delta R_{2DEG}(V_B, T)$, where V_B is the total DC bias applied to the circuit, I_{DS} is the current, and $\Delta R_{2DEG}(V_B, T)$ is a change in multiplexer resistance due to the potential energy in the GaAs 2DEG changing relative to the addressing gate voltages as a DC bias is applied. This term is not included in the definition of R_S introduced previously to analyse data without a DC bias applied (Figure S1). It describes an effective gating of the multiplexer due to V_B, which can either be accounted for during post-processing of data (as done here), or removed during the measurement itself by maintaining a fixed potential difference between addressing gates and the 2DEG.[15] It manifests as an approximately linearly decreasing background in measured resistance R as V_B increases (V_B is swept from -10 to 10 mV for all measurements), and is estimated as $\Delta R_{2DEG}(V_B, T) = R(-10, T) - R(10, T)$ where $R(-10, T)$ and $R(10, T)$ are the T-dependent resistance values at $V_B = -10$ and 10 mV, respectively. There is no apparent correlation with V_G, therefore we take the average ΔR_{2DEG} from measurements at different V_G for each T; $\Delta R_{2DEG} = \pm$ 10.5, 7.0, 7.1, 7.2, 7.1, 6.7, 6.2, and 5.6 kΩ for $T = 0.29$, 2, 5, 8, 11.2, 14.1, 17.8, and 25 K, respectively, with standard deviations $\sigma = 2.1$, 1, 1.1, 1.1, 1.1, 0.9, 0.7, and 0.5 kΩ. For comparison, total $R \gtrapprox 200$ kΩ.

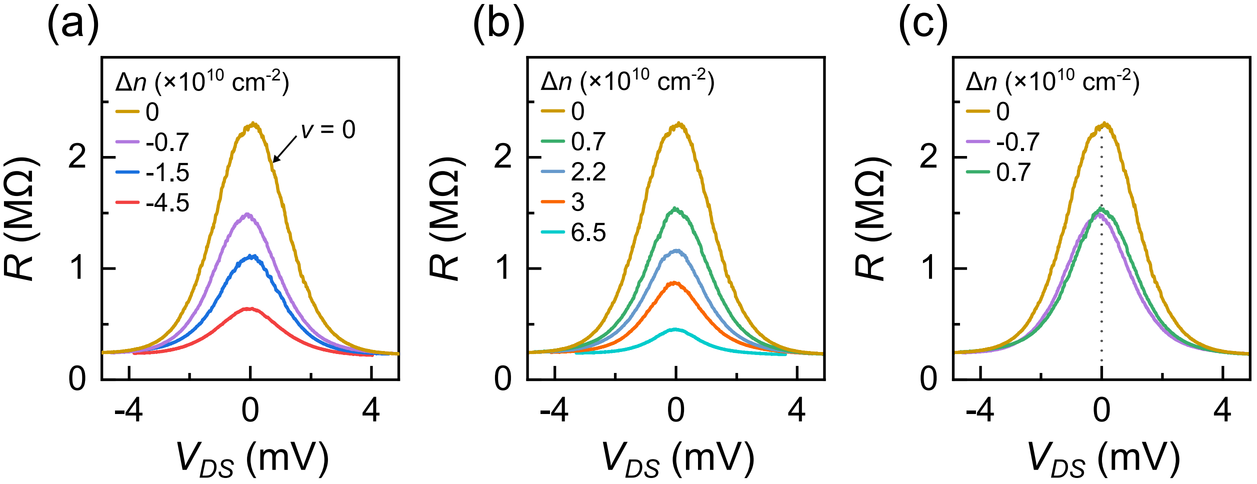

Figure S4: DC source–drain bias measurements at $B = 10$ T and $T \approx 1.4$ K. Data representing the $\nu = 0$ insulating state are obtained at $V_G = 0.32$ V, and figure legends are given in terms of $\Delta n = C_G(V_G - 0.32)/e$. (a and b) Resistance R as a function of DC source–drain bias V_{DS}. (a) and (b) show data on either side of the $\nu = 0$ insulating state, at $V_G \leq 0.32$ V and $V_G \geq 0.32$ V, respectively. The trace corresponding to $\nu = 0$ is labeled in (a). (c) Resistance R as a function of V_{DS} at the $\nu = 0$ insulating state and for n on either side. The vertical dotted line shows $V_{DS} = 0$ V.

Effective gating of the graphene due to source–drain bias. Figure S4(a) and (b) show resistance R prior to subtracting R_S as a function of source–drain bias across the graphene V_{DS} at back gate voltages V_G on either side of the $\nu = 0$ resistance peak at $B = 10$ T. Data representing $\nu = 0$ are obtained at $V_G = 0.32$ V, where R is determined to be maximum for this measurement when $V_{DS} = 0$ V. Panels (a) and (b) show data for $V_G \leq 0.32$ V and $V_G \geq 0.32$ V, respectively, and figure legends are defined using $\Delta n = C_G(V_G - 0.32)/e$. Measurements are performed at $T \approx 1.4$ K. There is a 50 mK temperature drift from $T = 1.46$ to 1.41 K between $V_G = 0.2$ and 0.5 V data, which has negligible effect on results. The V_{DS} is applied to the drain contact such that positive (negative) V_{DS} lowers (raises) the drain chemical potential, causing an effective p- (n-)doping of the graphene. For $V_G < 0.32$ V ($V_G > 0.32$ V) a positive V_{DS} reduces (increases) the graphene resistance, and negative V_{DS} increases (reduces) the graphene resistance, producing a slight asymmetry about $V_{DS} = 0$ V. Curves for $V_G < 0.32$ V, Figure S4(a), may appear slightly offset to the left with a shallower

(steeper) gradient for $V_{DS} < 0$ ($V_{DS} > 0$). The reverse is true for $V_G > 0.32$ V, Figure S4(b). At charge neutrality, positive and negative V_{DS} both reduce the graphene resistance. The asymmetry is further illustrated in Figure S4(c), which shows data on both sides of the resistance peak at $\nu = 0$ on a single plot. Differences between the peak heights of $\Delta n = \pm 0.7$ $\times 10^{10}$ cm^{-2} data at $V_{DS} = 0$ V arise from asymmetries in transfer characteristics.

This effective gating of the graphene due to V_{DS} is estimated as an equivalent change in V_G using[?] $E/v_F^2 = (h^2 n/4\pi v_F^2)^{1/2}$, where h is Planck's constant. Assuming V_{DS} is dropped linearly across the device, replacing E with an effective change in chemical potential in the graphene $eV_{DS}/2$, and n with $\Delta n = C\Delta V_G/e$ gives $|\Delta V_G| \approx 0.1$ mV at $V_{DS} \pm 1.5$ mV. This V_{DS} corresponds to the approximate full width at half maximum of V_{DS} curves at $T = 0.29$, 1.4, and 2 K, Figure 3(b) (main article) and Figure S4(a)–(c). The change in device resistance ΔR due to this effective gating depends on V_G. Using the maximum slope of the transfer curve R *vs* V_G (Figure 2(a), main article), to estimate the largest possible ΔR at $B = 10$ T gives $\Delta R \approx 10$ kΩ for an effective gating of 0.1 mV, which is $\approx 0.2\%$ of the measured R at this point. This gives a conservative upper bound for ΔR since the gradient is shallower near $V_G = 0.32$ V, the region of interest. This analysis uses data at $T = 0.29$ K. At higher T the transfer curve becomes shallower, which will reduce ΔR. Additionally, bias induced transport reduces the graphene resistance as V_{DS} is applied. The transfer curve used for this analysis is measured at $V_{DS} = 0$ V, and becomes shallower for finite $V_{DS} = 0$ V, further reducing ΔR from the effective gating. Therefore, although effective gating increases at high bias, *e.g.*, $\Delta V_G \approx 0.8$ mV at $V_{DS} = 4$ mV, its impact is limited by the lower $\Delta R/\Delta V_G$.

DC current–voltage characteristics for negative V_{DS}. Figure S5 (c)–(h) show DC current I_{DS} at negative V_{DS}. Positive V_{DS} data are plotted in Figure 4(c)–(h), main article. Positive and negative V_{DS} data show similar results, which suggests the effect of any asymmetry in the barrier shape in the tunneling region is small.

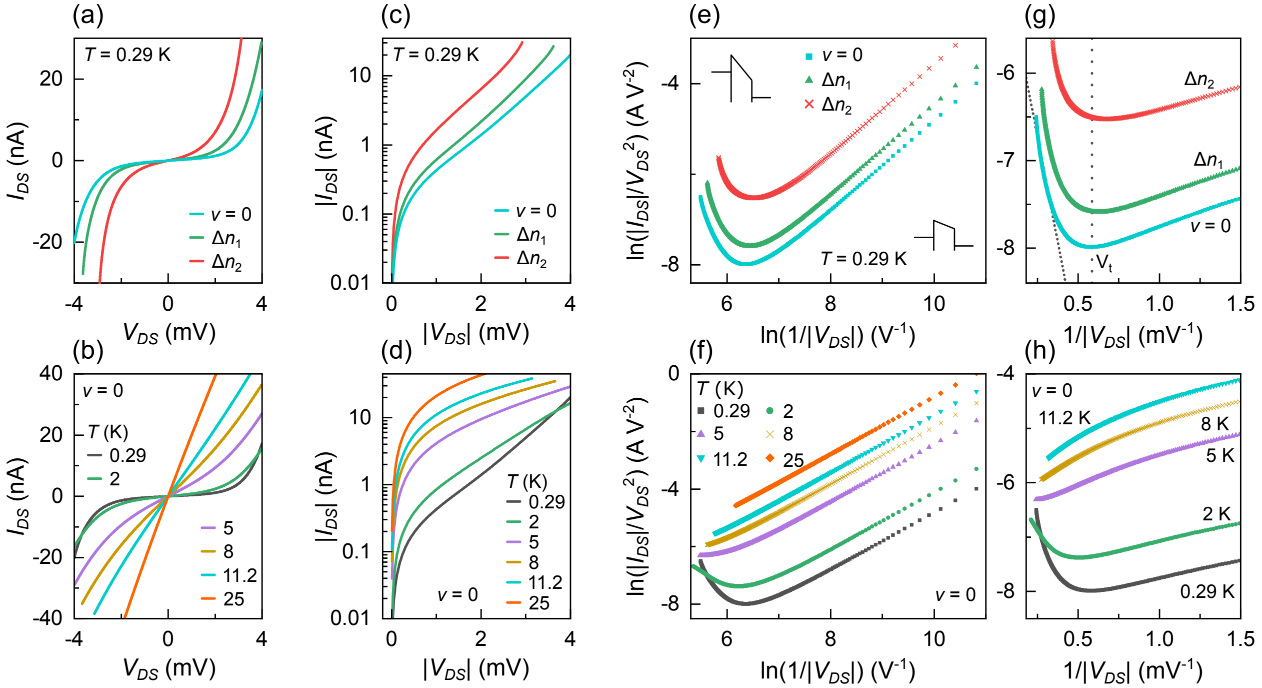

Figure S5: DC current-voltage characteristics for negative V_{DS}. Positive V_{DS} data are shown in Figure 4, main article. Magnetic field $B = 10$ T for all data in (a)–(h). The top row (a), (c), (e), and (g) show data at $\nu = 0$, $\Delta n_1 = -2.2\times10^{10}$ cm^{-2}, and $\Delta n_2 = -4.5\times10^{10}$ cm^{-2}, for $T = 0.29$ K. Data representing the $\nu = 0$ insulating state are obtained at $V_G = 0.32$ V, and $\Delta n = C_G(V_G - 0.32)/e$. The bottom row (b), (d), (f), and (h) show the T-dependence at $\nu = 0$. (a and b) Current I_{DS} as a function of source–drain bias across the graphene device V_{DS}. (c and d) Log-lin plots of $|I_{DS}|$ as a function of $|V_{DS}|$. (e and f) $\ln(|I_{DS}|/V_{DS}^2)$ as a function of $\ln(1/|V_{DS}|)$. Schematic diagrams in (e) represent the barrier shape in different bias regimes. (g and h) Fowler-Nordheim plots of $\ln(|I_{DS}|/V_{DS}^2)$ as a function of $1/|V_{DS}|$. The vertical dotted line labeled V_t in (g) corresponds to the inflection point voltage for $\nu = 0$ data, marking a transition between Fowler-Nordheim and direct tunneling. The diagonal dotted line shows a linear fit at high bias for Fowler-Nordheim tunneling.

Energy loss rates and carrier temperature. The differential conductance of the multiplexer and graphene in series is measured using an AC excitation voltage of $V_{AC} = 400$ μV due to the appreciable series resistance. The total power dissipated in the device is calculated using $P_{total} = V^2/R$, where V is the estimated AC voltage across the graphene accounting for series resistance, and R is resistance of the graphene. This gives $P_{total} < 0.2$ pW, and energy loss rate per carrier $P < 1.5\times10^{-18}$ W at $B = 10$ T, where $P = P_{total}/nA$, A is the device area and n is the carrier density. Parameters P_{total} and P are largest around charge neutrality. When calculating P the minimum carrier density is set to n_0, where

$n_0 = 1.3\ \times 10^{11}$ cm^{-2} is the residual carrier density from the main article. Both P_{total} and P reduce significantly at higher densities, for example $P \approx 2.5 \times 10^{-19}$ W for $n = 2\ \times 10^{11}$ cm^{-2} at $B = 10$ T. Using a $P = \alpha(n)T^4$ dependence reported for graphene,[16] extrapolating to low T, and taking $\alpha(n) \sim 1.4 \times 10^{-17}$ W K^{-4} per carrier from Figure 8 of Ref.[16] at $n = 1.3\ \times 10^{11}$ cm^{-2} (*i.e.*, n_0 for this measurement), gives $T < 0.6$ K. The residual carrier density n_0 is used since P is maximum around the charge neutrality point. At low B the reduced graphene resistance leads to higher dissipated power at charge neutrality, for example, at $B = 0$ T the maximum $P_{total} \lessapprox 2.1$ pW and $P \lessapprox 1.7 \times 10^{-17}$ W, corresponding to a maximum carrier temperature ~ 1 K.

Changing V_{AC} from 20 to 500 μV at $B = 10$ T and sweeping the back gate V_G for another device in the array showed no discernible change in the appearance of Landau levels, except for improved signal to noise. Measurements are performed at $T = 0.29$ K. A broadening of Landau levels might be expected if the carrier temperature had increased.[17] A different device (without a narrow constriction), is used in this study since several Landau levels could be more easily compared in a single gate sweep as R did not become insulating at charge neutrality. The energy loss rates estimated at $B = 10$ T are similar to results for the device with the narrow constriction, here the maximum $P_{total} \sim 0.14$ pW and maximum $P \sim 1.1 \times 10^{-18}$ W, respectively, corresponding to a carrier temperature < 0.6 K.

Raman spectroscopy. Raman spectra are measured in a $10 \times 10\ \mu$m^2 grid over the graphene area, with a grid spacing of 1 μm. Figure S6(a) shows an example of the background-subtracted 2D and G peaks[18] from the area around the narrow constriction. A map of the 2D to G peak ratio is shown in Figure S6(b), where the 2D to G peak ratio 2D/G > 1 is consistent with monolayer graphene. Data in Figure S6(a) correspond to location $(6, 9)$, where coordinates refer to the x and y locations numbered from $(1, 1)$, bottom left. The 1 μm laser spot size causes broadening of the image compared to the scanning electron microscopy image in Figure 1(a), main article. The peak intensities shown in Figure S6(b)

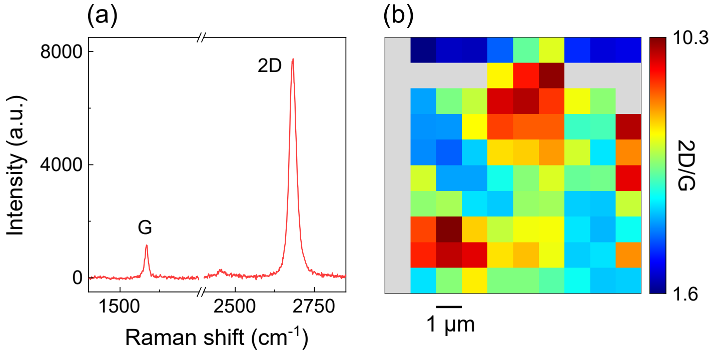

Figure S6: Raman spectra. (a) Example Raman spectra around the narrow constriction in the graphene. Data are from cell (6,9), referring to the x and y locations referenced from the bottom left cell (1,1) in (b). (b) Mapping of the 2D to G peak ratio 2D/G for a 10 μm^2 area over the graphene device, with a 1 μm grid spacing. The grey color corresponds to where peaks cannot be determined.

are found by extracting the maximum intensities for ranges around the 2D and G peaks, after removing a background. The grey squares represent when a peak is not detected within the noise of the measurement for either the G or 2D range.

Methods for the deliberately–defined constriction. A non–multiplexed, two–terminal device with the same source–drain contact configuration as the multiplexed device is fabricated on a doped Si wafer with 285 nm–thick thermally grown SiO_2 as a global back–gate dielectric. Source–drain contacts (Ti/Au) are deposited prior to wet transfer of monolayer CVD graphene. A $\sim$ 50 nm Al_2O_3 encapsulating layer is added by atomic layer deposition (ALD) after patterning.[19] Measurements are performed in a cryostat with a base $T =\sim 1.5$ K, and resistance is measured using a constant current lock–in technique with $I = 100$ nA at 17 Hz.

The transfer curve (red) in Figure S7 measured at $B = 0$ T and $T = 1.5$ K shows two resistance peaks labelled a and b, separated by $V_G \sim 20$ V. We fit these two peaks with eq 1 (main article), which separates the graphene channel into two resistive components in series with distinct lithographically–defined aspect ratios. We use $(L/W)_a = 3$ and $(L/W)_b = 1$ to represent the constriction and main channel, respectively. The black curve shows the

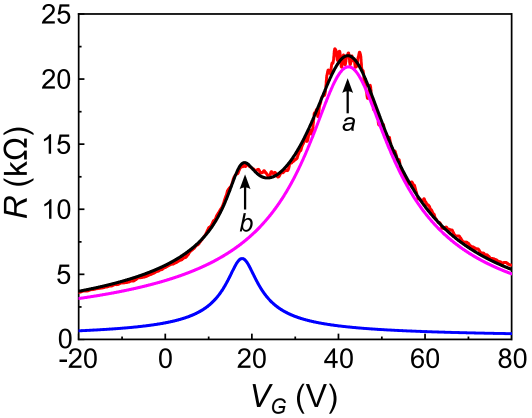

Figure S7: Transfer characteristics for the deliberately–defined constriction. The red curve shows R as a function of back gate voltage at $B = 0$ T and $T = 1.5$ K. The magenta and blue curves shows fits for peaks a and b, respectively, using eq 1, main article. The black curve shows the combined fit.

addition of the two, plus parasitic resistance R_P. Peak fitting estimates mobility $\mu_a = 1290$ cm^2 V^{-1} s^{-1} and residual carrier density $n_{0,a} = 0.7 \times 10^{12}$ cm^{-2} for peak a, compared to $\mu_b = 3890$ cm^2 V^{-1} s^{-1} and $n_{0,b} = 0.26 \times 10^{12}$ cm^{-2} for peak b, with $R_P \approx 100$ Ω. These values are very approximate since L/W ratios are not well defined, and this fitting is merely provided to illustrate how the larger L/W ratio of the constriction is consistent with peak a and lower L/W ratio of the wide channel with peak b. We also note that the additional p–doping (*i.e.*, the positive shift in V_G compared to peak b), the larger residual carrier density, and the lower mobility of peak a are expected due to the oxygen plasma etching process and enhanced impact of the doped etched edges on transport through the constriction relative to the wide section. The separation of the two peaks in V_G allows us to view the temperature and magnetic field dependent behavior of the constriction and wide area separately.

REFERENCES

(1) Kim, S.; Nah, J.; Jo, I.; Shahrjerdi, D.; Colombo, L.; Yao, Z.; Tutuc, E.; Banerjee, S. K. Realization of a High Mobility Dual-Gated Graphene Field-Effect Transistor With Al_2O_3 Dielectric. *Applied Physics Letters* **2009**, *94*, 062107.

(2) Smith, L. W.; Batey, J. O.; Alexander-Webber, J. A.; Fan, Y.; Hsieh, Y.-C.; Fung, S.-J.; Jevtics, D.; Robertson, J.; Guilhabert, B. J. E.; Strain, M. J.; Dawson, M. D.; Hurtado, A.; Griffiths, J. P.; Beere, H. E.; Jagadish, C.; Burton, O. J.; Hofmann, S.; Chen, T.-M.; Ritchie, D. A.; Kelly, M. *et al.* High-Throughput Electrical Characterization of Nanomaterials from Room to Cryogenic Temperatures. *ACS Nano* **2020**, *14*, 15293–15305.

(3) Zhong, H.; Zhang, Z.; Xu, H.; Qiu, C.; Peng, L.-M. Comparison of Mobility Extraction Methods Based on Field-Effect Measurements for Graphene. *AIP Adv.* **2015**, *5*, 057136.

(4) Kurganova, E. V.; Giesbers, A. J. M.; Gorbachev, R. V.; Geim, A. K.; Novoselov, K. S.; Maan, J. C.; Zeitler, U. Quantum Hall Activation Gaps in Bilayer Graphene. *Solid State Communications* **2010**, *150*, 2209–2211.

(5) Chiappini, F.; Wiedmann, S.; Novoselov, K.; Mishchenko, A.; Geim, A. K.; Maan, J. C.; Zeitler, U. Lifting of the Landau Level Degeneracy in Graphene Devices in a Tilted Magnetic Field. *Phys. Rev. B* **2015**, *92*, 201412.

(6) Veyrat, L.; Déprez, C.; Coissard, A.; Li, X.; Gay, F.; Watanabe, K.; Taniguchi, T.; Han, Z.; Piot, B. A.; Sellier, H.; Sacépé, B. Helical Quantum Hall Phase in Graphene on $SrTiO_3$. *Science* **2020**, *367*, 781.

(7) Young, A. F.; Dean, C. R.; Wang, L.; Ren, H.; Cadden-Zimansky, P.; Watanabe, K.; Taniguchi, T.; Hone, J.; Shepard, K. L.; Kim, P. Spin and Valley Quantum Hall Ferromagnetism in Graphene. *Nature Physics* **2012**, *8*, 550–556.

(8) Checkelsky, J. G.; Li, L.; Ong, N. P. Divergent Resistance at the Dirac Point in Graphene: Evidence for a Transition in a High Magnetic Field. *Phys. Rev. B* **2009**, *79*, 115434.

(9) Giesbers, A. J. M.; Ponomarenko, L. A.; Novoselov, K. S.; Geim, A. K.; Katsnel-

son, M. I.; Maan, J. C.; Zeitler, U. Gap Opening in the Zeroth Landau Level of Graphene. *Phys. Rev. B* **2009**, *80*, 201403.

(10) Du, X.; Skachko, I.; Duerr, F.; Luican, A.; Andrei, E. Y. Fractional Quantum Hall Effect and Insulating Phase of Dirac Electrons in Graphene. *Nature* **2009**, *462*, 192–195.

(11) Bolotin, K. I.; Ghahari, F.; Shulman, M. D.; Stormer, H. L.; Kim, P. Observation of the Fractional Quantum Hall Effect in Graphene. *Nature* **2009**, *462*, 196–199.

(12) Giesbers, A. J. M.; Zeitler, U.; Katsnelson, M. I.; Ponomarenko, L. A.; Mohiuddin, T. M.; Maan, J. C. Quantum-Hall Activation Gaps in Graphene. *Phys. Rev. Lett.* **2007**, *99*, 206803.

(13) Zhang, Y.; Jiang, Z.; Small, J. P.; Purewal, M. S.; Tan, Y.-W.; Fazlollahi, M.; Chudow, J. D.; Jaszczak, J. A.; Stormer, H. L.; Kim, P. Landau-Level Splitting in Graphene in High Magnetic Fields. *Phys. Rev. Lett.* **2006**, *96*, 136806.

(14) Foa Torres, L. E. F.; Roche, S.; Charlier, J.-C. *Introduction to Graphene-Based Nanomaterials: From Electronic Structure to Quantum Transport*; Cambridge University Press: Cambridge, U.K., 2014.

(15) Yi, T. Progress Towards GaAs Multiplexed Single-Electron Pump Arrays. Ph.D. thesis, University of Cambridge, 2019.

(16) Baker, A. M. R.; Alexander-Webber, J. A.; Altebaeumer, T.; McMullan, S. D.; Janssen, T. J. B. M.; Tzalenchuk, A.; Lara-Avila, S.; Kubatkin, S.; Yakimova, R.; Lin, C.-T.; Li, L.-J.; Nicholas, R. J. Energy Loss Rates of Hot Dirac Fermions in Epitaxial, Exfoliated, and CVD Graphene. *Phys. Rev. B* **2013**, *87*, 045414.

(17) Funk, H.; Knorr, A.; Wendler, F.; Malic, E. Microscopic View on Landau Level Broadening Mechanisms in Graphene. *Phys. Rev. B* **2015**, *92*, 205428.

(18) Ferrari, A. C.; Basko, D. M. Raman Spectroscopy as a Versatile Tool for Studying the Properties of Graphene. *Nature Nanotechnology* **2013**, *8*, 235–246.

(19) Alexander-Webber, J. A.; Sagade, A. A.; Aria, A. I.; Van Veldhoven, Z. A.; Braeuninger-Weimer, P.; Wang, R.; Cabrero-Vilatela, A.; Martin, M.-B.; Sui, J.; Connolly, M. R.; Hofmann, S. Encapsulation of Graphene Transistors and Vertical Device Integration by Interface Engineering with Atomic Layer Deposited Oxide. *2D Mater.* **2017**, *4*, 011008.
